# Learning about and from others' prudence, impatience or laziness: The computational bases of attitude alignment

**DOI:** 10.1371/journal.pcbi.1005422

**Published:** 2017-03-30

**Authors:** Marie Devaine, Jean Daunizeau

**Affiliations:** 1 Brain and Spine Institute (ICM), Paris, France; 2 ETH, Zurich, Switzerland; University College London, UNITED KINGDOM

## Abstract

Peoples' subjective attitude towards costs such as, e.g., risk, delay or effort are key determinants of inter-individual differences in goal-directed behaviour. Thus, the ability to learn about others' prudent, impatient or lazy attitudes is likely to be critical for social interactions. Conversely, how adaptive such attitudes are in a given environment is highly uncertain. Thus, the brain may be tuned to garner information about how such costs ought to be arbitrated. In particular, observing others' attitude may change one's uncertain belief about how to best behave in related difficult decision contexts. In turn, learning *from* others' attitudes is determined by one's ability to learn *about* others' attitudes. We first derive, from basic optimality principles, the computational properties of such a learning mechanism. In particular, we predict two apparent cognitive biases that would arise when individuals are learning about others’ attitudes: (i) people should overestimate the degree to which they resemble others (false-consensus bias), and (ii) they should align their own attitudes with others’ (social influence bias). We show how these two biases non-trivially interact with each other. We then validate these predictions experimentally by profiling people's attitudes both before and after guessing a series of cost-benefit arbitrages performed by calibrated artificial agents (which are impersonating human individuals).

## Author summary

What do people learn *from* observing others' attitudes, such as "prudence", "impatience" or "laziness"? Rather than viewing these attitudes as examples of highly subjective personality traits, we assume that they derive from uncertain (and mostly implicit) beliefs about how to best weigh risks, delays and efforts in ensuing cost-benefit trade-offs. In this view, it is adaptive to update one's belief after having observed others' attitude, which provides valuable information regarding how to best behave in related difficult decision contexts. This is the starting point of our computational model of attitude alignment, which we derive from first optimality principles as well as from recent neuroscientific findings. Critical here is the impact of one's ability to learn *about* others' covert mental states or attitudes, which is known as "mentalizing" or "Theory of Mind". In particular, this model makes two (otherwise unrelated) predictions that conform to known but puzzling cognitive biases of social cognition in humans, namely: "false consensus" and "social influence". It also shows how attitude alignment may eventually follow from the interaction between these two biases. Using state-of-the-art behavioural and computational methods, we provide experimental evidence that confirm these predictions. Finally, we discuss the relevance and implications of this work, both from a neuroscientific and economic perspective.

## Introduction

Subjective traits or attitudes such as "prudence", "impatience" or "laziness" are key determinants of goal-directed behaviour. This is because they determine how people arbitrate between canonical but conflicting decision dimensions, e.g., the prospect of reward and costs such as risk, delay or effort [1–3]. For example, high risk devaluation is the hallmark of "prudence", "impatience" is associated with strong delay discounting and "lazy" people find potential rewards not worth the effort. Importantly, although some variant of these phenotypes may eventually prove to have higher adaptive fitness [4,5], our socio-ecological niche only provides sparse and ambivalent feedback regarding how prudent, impatient or lazy one should be [6,7]. This typically results in high uncertainty regarding how to best behave in related difficult decision contexts, which may be why we are influenced by others' attitude [8,9]. But is the brain tuned to garner such information from others, even when this is not directly instrumental? Does this non-trivially depend upon our ability to learn about others’ covert attitudes? Reciprocally: do our own attitude impacts on how we learn about others'? Also: given that such cognitive mechanism may have been optimized through natural selection, can we derive its computational bases from first optimality principles? These are the questions we address in this work, using a combination of experimental and computational methods.

Recall that humans seem to engage in imitative behaviour automatically, even in the absence of incentives or social norms [10]. For example, it has been shown that people tend to imitate others' motor actions [11] and mirror their emotions [12] when this is not instrumental to the task, or even when this compromises the efficiency of goal-directed behaviour [13]. Such social influence also applies to overt motives, giving rise to phenomena such as "goal contagion" [14,15] and “mimetic desires” [16,17], whereby objects’ attractiveness increases when they are wanted by other peoples. Recent neuroscientific investigations of this "influence bias" have disclosed an intriguing form of biological determinism. First, social influence disrupts the neural computation of subjective values that takes place in the so-called "brain valuation system" or BVS [8,18]. Second, the magnitude of the influence bias is predicted by the coupling strength between the BVS and the brain’s “mirror neuron system” [19]. Recall that the latter has been repeatedly shown to play a major role in action understanding for a wide range of animal species, from birds [20] to monkeys [11] to humans [21,22]. In humans, it is known to be involved whenever people engage in "mentalizing" or "Theory of Mind" (ToM), i.e. interpretations of others' overt behaviour in terms of (covert) mental states or attitudes [23,24]. These findings are important because they suggest that mentalizing about others' attitudes may automatically (although maybe implicitly) trigger the alignment of the observer's attitude towards the observee's.

The conventional view here is that such attitude alignment should be considered a cognitive bias, which may inherit its adaptive fitness from the fact that it facilitates social conformity [25,26]. However, evolutionary models have long shown that selective pressure may eventually favour imitative phenotypes, even when conformity brings no survival and/or mating advantage [27–29]. This is because imitation is a form of fast learning (from others), which essentially circumvents the necessity to rediscover utile information [30,31]. In line with these ideas, we start with the premise that subjective attitudes such as "prudence", "impatience" or "laziness" may be best understood as uncertain (and mostly implicit) beliefs about how to “best” weigh risks, delays and efforts, respectively. From an information-theoretic perspective, this implies that these beliefs can be updated, given new information about how costs and benefits ought to be arbitrated. Under this view, a mentalizing agent who would not align her attitude with others' would be suboptimal, because she would essentially neglect relevant information. Critical here is the fact that such subjective attitudes are not directly accessible: they typically have to be inferred from overt behaviour. This implies that attitude alignment may be non-trivially shaped by the computational properties of mentalizing. For example, people tend to overestimate the degree to which they resemble others, which is known as the "false consensus" bias [32,33]. In this work, we derive a Bayesian model of attitude alignment that predicts, from first (evolutionary) principles, the existence of false-consensus and influence biases as well as their non-trivial interaction. We then validate these predictions experimentally by profiling people's attitudes both before and after guessing a series of cost-benefit arbitrages performed by calibrated artificial agents (which are impersonating human individuals).

This paper is organized as follows. The first section of our manuscript exposes our computational and empirical methods. First, we recall how subjective traits or attitudes such as "prudence", "impatience" or "laziness" relate to overt cost-benefit arbitrages. Second, we extend existing Bayesian models of mentalizing [34–37] to the problem of learning such subjective attitudes. Third, we summarize the derivation of our computational model of attitude alignment, from which we disclose predictions that can be tested empirically. The second section of our manuscript summarizes our experimental results, using an experimental design inspired from behavioural economics choice paradigms that aim at revealing people’s attitude towards delay, effort and risk. In particular, we ran three series of statistical data analysis of increasing computational sophistication. The last section of the manuscript consists of a discussion of the limitations of our study, in the context of the existing literature.

## Methods

### Ethics statement

This study has been approved by the local ethics' committee (CPP-Ile-de-France 1) as part of a larger research program, and was conducted according to the principles expressed in the Declaration of Helsinki. Written informed consent was obtained from the participants.

### 1. Computational modelling

Our quantitative modelling strategy is twofold, i.e. we have to consider (i) models of participants' cost-benefit arbitrages in the *Decision* phases (these will also be used to simulate artificial agents' behaviour in the *Prediction* phase), and (ii) models of how people learn about and from others' choices. The former stem from classical decision theory and are borrowed from behavioural economics. They assume that decisions follow from a comparison between the value or utility of each alternative option. The latter are an instance of the meta-Bayesian approach [38,39], which essentially is an information theoretic perspective on inferring how agents make decisions under uncertainty. They describe mentalizing by embedding the former cost-benefit arbitrage models into a trial-by-trial Bayesian belief update scheme.

#### 1.1. Modelling cost-benefit arbitrages

Our use of decision models is threefold: (i) to profile participants’ attitudes from their choices in the *Decision* phases, (ii) to simulate artificial agents' choices in the *Prediction* phase, and (iii) to serve as prior assumptions for mentalizing models in the *Prediction* phase (see below). In what follows, behavioural observations consist in binary choices between two alternatives that differ along two dimensions, namely expected reward and cost (delay, effort or risk). According to decision theory, we assume that the probability of choosing a given option increases with its subjective value or utility. More precisely, the probability *P*_1_ of choosing option 1 (over option 2) is given by the following probabilistic, softmax rule [40]:

$$P_{1}=\frac{\exp\left(\beta \;V_{1}\right)}{\exp\left(\beta \;V_{1}\right)+\exp\left(\beta \;V_{2}\right)}$$ (1)

where *V*_*i*_ is the subjective value of option *i*, and *β* is a behavioural inverse-temperature that controls the magnitude of potential deviations from rationality (at the limit *β* → ∞, Eq 1 reduces to the utility-maximization deterministic policy).

Let Op_*i*_ be the *i*^th^ alternative option, which is defined in terms a given reward/cost duplet (e.g., for inter-temporal choices: Op_1_ = "1€ now" and Op_2_ = "10€ in a week"). The ensuing cost-benefit arbitrage is controlled by people's susceptibility to costs *α*, which parameterizes a utility function *u*_*α*_ (Op_*i*_) = *V*_*i*_. More precisely, the mathematical form of utility functions is cost-dependent:

- *Delay*: the utility of a payoff *R* obtained after a delay *T* is given by the hyperbolic utility function [41]:
  $$u_{\alpha }\left(R,T\right)=\frac{R}{1+\alpha T}$$ (2)
  where *α* controls people's susceptibility to delay. Here delay induces a divisive cost, which implies that one would always prefer a delayed reward to nothing now.
- *Risk*: the utility of obtaining a payoff *R* with probability *P* is given by the following exponential utility function:
  $$u_{\alpha }\left(R,P\right)=P\left(1-\exp\left(-R\alpha \right)\right)$$ (3)
  where *α* controls the concavity of the utility function, and hence people's susceptibility to risk (Pratt, 1964). In this case, a sure reward may eventually be preferable to a higher but risky outcome.
- *Effort*: the utility of a payoff *R* obtained when exerting an effort *E* is given by the following effort-discounting utility function:
  $$u_{\alpha }\left(R,E\right)=R-\alpha \frac{E}{1-E}$$ (4)
  where *α* controls people's susceptibility to effort. Note that effort *E* is defined in terms of the percentage of the individual's maximal force (physiological limit). This utility function has the desirable property that the cost of a supplementary unit of Effort increases as the Effort gets closer to the participant’s physiological limit [42].

Inserting Eqs 2–4 into Eq 1 (i.e. defining *V*_*i*_ ≜ *u*_*α*_ (Op_*i*_)) effectively models cost-benefit arbitrages in terms of a comparison between alternative options' utilities. These cost-benefit arbitrage models have two free parameters, namely: the cost-susceptibility *α* and the behavioural inverse-temperature *β*. In particular, the subjective aspect of these arbitrages is captured by the cost-susceptibility parameter *α*, which determines how impatient, prudent or lazy people's overt behaviour is. For example, a high delay-susceptibility induces strong temporal discounting, eventually yielding impatient behaviour (i.e. a preference towards short-delay options). Similarly, high risk (resp. effort) susceptibility yields prudent (resp. lazy) behaviour. In what follows, we will thus refer to *α* as a covert subjective attitude or trait. In the next section, we will see how mentalizing on such attitudes can be modelled in terms of a Bayesian belief update scheme.

#### 1.2. The Bayesian preference learner

Let us now introduce a Bayesian model of how people update their belief about others' impatient, lazy or prudent attitude, under the so-called "rationality principle" [36,43–45]. In our case, this reduces to estimating the relevant cost-susceptibility parameter *α* (as well as the behavioural temperature *β*) from observed choices.

First, mentalizing Bayesian agents assume that the *Other*'s choices obey the softmax decision rule of Eq 1. This yields the following binomial likelihood *p*(*a*_→*t*_|*θ*^(*o*)^) for the Other's decision *a*_→*t*_ (up to trial *t*):

$$\begin{array}{l} p\left(a_{\to t}|\theta^{\left(o\right)}\right)=\prod_{t'=1}^{t}p\left(a_{t'}|\theta^{\left(o\right)}\right)\\ \;p\left(a_{t}|\theta^{\left(o\right)}\right)=P_{1}{}^{a_{t}}{\left(1-P_{1}\right)}^{1-a_{t}} \end{array}$$ (5)

where *a*_*t*_ ∈ {0,1} is the Other's binary choice at trial *t*, *P*_1_ ≜ *p*(*a*_*t*_ = 1|*θ*^(*o*)^) is the probability that the Other chooses the first alternative option (cf. Eq 1) and *θ*^(*o*)^ = {log *α*^(*o*)^,log *β*^(*o*)^} is the set of unknown parameters that summarizes the Other's covert attitude or trait. Note that Eq 5 holds, irrespective of the type of cost (i.e. delay, effort or risk). The mathematical form of the corresponding utility functions are given in Eqs 2, 3 and 4.

Second, before having observed any Other's decision, the agent is endowed with some prior belief *p*(*θ*^(*o*)^) about the Other's trait *θ*^(*o*)^. Here, we assume that this prior belief $p\left(\theta^{\left(o\right)}\right)=N\left(\mu_{0}^{\left(o\right)},\Sigma_{0}^{\left(o\right)}\right)$ is Gaussian with mean $\mu_{0}^{\left(o\right)}$ (which captures the direction of the agent's bias) and variance $\Sigma_{0}^{\left(o\right)}$ (which measures how uncertain is the agent's prior belief).

Observing the Other's choices gives the agent information about *θ*^(*o*)^, which can be updated trial after trial using the following Bayes-optimal probabilistic scheme:

$$\begin{array}{l} p\left(\theta^{\left(o\right)}|a_{\to t}\right)\propto p\left(a_{\to t}|\theta^{\left(o\right)}\right)p\left(\theta^{\left(o\right)}\right)\\ \;\propto p\left(a_{t}|\theta^{\left(o\right)}\right)p\left(\theta^{\left(o\right)}|a_{\to t-1}\right) \end{array}$$ (6)

where *p*(*θ*^(*o*)^|*a*_→*t*_) is the agent's posterior belief about the Other's trait after trial *t* and the second line highlights the sequential (online) form of Bayesian belief update. Practically speaking, we implement this learning model using a variational-Laplace scheme [46,47], which yields semi-analytical expressions for the update rules of the two first moments ($\mu_{t}^{\left(o\right)}$ and $\Sigma_{t}^{\left(o\right)}$) of the posterior belief $p\left(\theta^{\left(o\right)}|a_{\to t}\right)\approx N\left(\mu_{t}^{\left(o\right)},\Sigma_{t}^{\left(o\right)}\right)$. This completes our description of the Bayesian Preference Learner (BPL). We refer the interested reader to S1 Text for relevant mathematical details.

Note that the BPL model is entirely specified by the two first moments ($\mu_{0}^{\left(o\right)}$ and $\Sigma_{0}^{\left(o\right)}$) of the agent's prior belief. In particular, the prior variance $\Sigma_{0}^{\left(o\right)}$ controls the resilience of the agent's prior bias to observational information regarding the Other's trait. In other words, the smaller the prior variance $\Sigma_{0}^{\left(o\right)}$, the stronger the prior bias in his posterior belief. Should the prior mean $\mu_{0}^{\left(o\right)}$ match the agent's own attitude *θ*^(*s*)^ (i.e. when $\mu_{0}^{\left(o\right)}\approx \theta^{\left(s\right)}$), she would then exhibit a "perfect" false-consensus bias. We will introduce a normative model of this below. But let us first comment on one non-trivial computational property of the BPL model.

**Fig 1:**
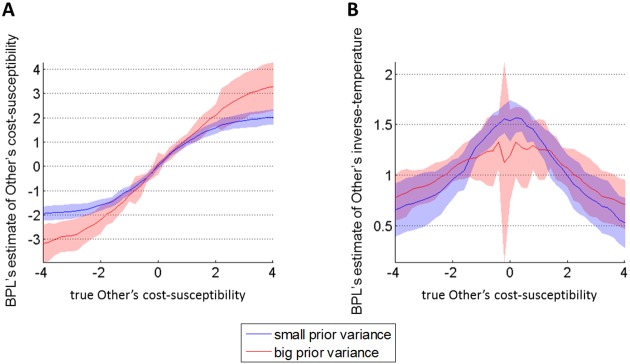
Response of the BPL model. We simulated virtual BPL learners equipped with neutral priors ($\mu_{0}^{\left(o\right)}=0$), who learned about agents (endowed with varying cost-susceptibilities) performing cost-benefit arbitrages (*T* = 40 choices, as in the main experiment). The Monte-Carlo average (plain lines) and standard deviations (shaded areas) of BPL's posterior estimates were obtained by repeating this simulation with random pairings of benefits and costs. We also varied BPL's prior variance on the Other's cost susceptibility (blue: small prior variance, red: high prior variance). **A:** BPL's posterior estimate of the Other's cost-susceptibility (y-axis) is plotted as a function of the true Other's cost-susceptibility (x-axis). **B**: BPL's posterior estimate of the Other's inverse-temperature (y-axis) is plotted as a function of the true Other's cost-susceptibility (x-axis).

Note that an observed choice that does not match the BPL's prediction can be explained away either by updating her posterior estimate of the Other's cost-susceptibility, or by increasing the posterior estimate of the temperature. In turn, the impact of the next prediction error will be smaller, i.e. it will induce a smaller update $\mu_{t}^{\left(o\right)}-\mu_{t-1}^{\left(o\right)}$. In other words, the rate of posterior update $\mu_{t}^{\left(o\right)}-\mu_{0}^{\left(o\right)}$ decreases as the true Other's cost-susceptibility *α*^(*o*)^ further departs from the prior mean $\mu_{0}^{\left(o\right)}$. The response of the BPL model to the true Other's cost-susceptibility *α*^(*o*)^ is summarized on Fig 1 below.

One can see that, for small *α*^(*o*)^, BPL's estimate of *α*^(*o*)^ tracks the true *α*^(*o*)^ in a linear fashion; however, as the true *α*^(*o*)^ increases, BPL's estimate of *α*^(*o*)^ tends to saturate (cf. Fig 1A). In turn, the global relationship between the two is sigmoidal, where the upper and lower bounds increase in magnitude with BPL's prior variance $\Sigma_{0}^{\left(o\right)}$. This is explained by the fact that BPL's estimate of *β*^(*o*)^ decreases as *α*^(*o*)^ increases in magnitude (cf. Fig 1B). In brief, strongly surprising observations are regarded as errors: they are deemed less informative than observations that better conform to BPL's prior guess. One can show that, at the uninformative prior limit (i.e. when $\Sigma_{0}^{\left(o\right)}\to \infty$), the BPL's posterior mean $\mu_{t}^{\left(o\right)}$ tends to the unbiased maximum-likelihood estimator. Thus, one may be tempted to think that any finite prior variance may eventually yield biased estimates, which would eventually degrade prediction performance. The latter reasoning is misguided however, due to the bias-variance trade-off of statistical learning [48]. This trade-off can be eyeballed on Fig 1A, by noting that the variance (across Monte-Carlo simulations) of BPL's posterior estimates increases with $\Sigma_{0}^{\left(o\right)}$. In fact, at the uninformative prior limit (and given limited data), BPL's posterior estimates would be so unreliable that the average generalization error would be maximal. Note that this holds true irrespective of the prior mean. This is a first hint of why the false-consensus bias can be deemed optimal from an information theoretic perspective. As we will see below, this computational property of Bayesian mentalizing eventually induces non-trivial limitations to attitude alignment.

#### 1.3. A Bayesian account of attitude alignment

We now revisit the computational bases of attitude alignment, taking inspiration from models of "informational cascades" (Shiller, 1995), which describe how imitative behaviour arise from implicit information sharing among agents. Recall that people's subjective attitudes can be seen as uncertain beliefs about "best" policies, where "best" is only defined implicitly (without explicit reference to an objective performance measure). In what follows, we show how prior assumptions regarding how information about the "best" policy is scattered across individuals eventually yields both false-consensus and influence biases, and how it ties them together.

To begin with, recall that "how good" attitudes towards effort, delay or risk is likely to be environment-dependent. For example, the optimal delay discounting depends upon the environmental hazard rate, which may be unknown [49]. Thus, let us assume that, in a given environment, there is an objective "best" policy *η* (i.e. a "best" way of discounting delay, effort or risk), which is hidden from agents but indirectly accessible through noisy (e.g., reinforcement) signals *y*^(*i*)^ = *η* + *ε*^(*i*)^, where $\varepsilon^{\left(i\right)}∼N\left(0,\sigma_{\varepsilon }^{\left(i\right)}\right)$ are i.i.d. gaussian random errors with variance $\sigma_{\varepsilon }^{\left(i\right)}$. For the sake of simplicity, we consider that the reinforcement error variance is identical across individuals, i.e.: $\sigma_{\varepsilon }^{\left(i\right)}≜\sigma_{\varepsilon }^{}$. This provides agents with a likelihood function *p*(*y*^(*i*)^|*η*,*σ*_*ε*_) regarding the "best" policy *η*. From a Bayesian perspective, individuals are endowed with an innate subjective prior $p\left(\eta |\eta_{G}^{\left(i\right)},\sigma_{G}\right)=N\left(\eta_{G}^{\left(i\right)},\sigma_{G}\right)$ about the best policy *η*, which we parameterize in terms of its mean $\eta_{G}^{\left(i\right)}$ and variance *σ*_*G*_. Without loss of generality, we assume that the innate prior means $\eta_{G}^{\left(i\right)}$ are scattered across individuals according to a Gaussian distribution with mean Γ_*G*_ and variance Ω_*G*_, i.e.: $\eta_{G}^{\left(i\right)}∼N\left(\Gamma_{G},\Omega_{G}\right)$. We will see that, taken together, these assumptions imply that mentalizing (Bayesian) agents are bound to false-consensus and influence biases.

But first, what can we say about the agent's own attitude *α*^(*i*)^? Over the agent's lifetime, her prior $p\left(\eta |\eta_{G}^{\left(i\right)},\sigma_{G}\right)$ is integrated with the likelihood *p*(*y*^(*i*)^|*η*,*σ*_*ε*_) to yield a posterior belief $p\left(\eta |y^{\left(i\right)},\sigma_{\varepsilon },\eta_{G}^{\left(i\right)},\sigma_{G}\right)≜N\left(\alpha_{}^{\left(i\right)},\sigma \right)$ regarding the "best" policy, whose mean is (by definition) the agent's attitude *α*^(*i*)^:

$$\left\{ \begin{array}{l} \alpha^{\left(i\right)}=\sigma \left(\frac{y^{\left(i\right)}}{\sigma_{\varepsilon }}+\frac{\eta_{G}^{\left(i\right)}}{\sigma_{G}}\right)\\ \sigma ={\left(\frac{1}{\sigma_{\varepsilon }}+\frac{1}{\sigma_{G}}\right)}^{-1} \end{array}\right.$$ (7)

where *σ* is the agent's uncertainty regarding the "best" policy. Eq 7 holds for any agent, but eventually yields different traits *α*^(*i*)^, owing to different feedback errors *ε*^(*i*)^ and different prior means $\eta_{G}^{\left(i\right)}$. In what follows, *α*^(*s*)^ (resp., *α*^(*o*)^) will denote the agent's (resp., the Other's) trait.

At this point, the agent's prior belief *p*(*α*^(*o*)^|*α*^(*s*)^,*σ*_*ε*_,*σ*_*G*_,Γ_*G*_,Ω_*G*_) regarding the Other's trait *α*^(*o*)^ (cf. BPL model above) can now be derived in terms of a prediction about others' subjective estimate of the "best" policy:

$$\begin{array}{c} p\left(\alpha^{\left(o\right)}|\alpha^{\left(s\right)},\sigma_{\varepsilon },\sigma_{G},\Gamma_{G},\Omega_{G}\right)=\iint p\left(\alpha^{\left(o\right)}|\eta ,\sigma_{\varepsilon },\sigma_{G},\eta_{G}^{\left(o\right)}\right)p\left(\eta |\alpha^{\left(s\right)},\sigma \right)p\left(\eta_{G}^{\left(o\right)}|\Gamma_{G},\Omega_{G}\right)\;d\mu \;d\eta_{G}^{\left(o\right)}\\ ≜N\left(\mu_{0}^{\left(o\right)},\Sigma_{0}^{\left(o\right)}\right) \end{array}$$ (8)

where $p\left(\alpha^{\left(o\right)}|\eta ,\sigma_{\varepsilon },\sigma_{G},\eta_{G}^{\left(o\right)}\right)$ derives from Eq 7 (having replaced the other's signal *y*^(*o*)^ with its definition (i.e.: *y*^(*o*)^ = *η* + *ε*^(*o*)^), and $p\left(\eta |\alpha^{\left(s\right)},\sigma_{}^{\left(s\right)}\right)≜p\left(\eta |y^{\left(s\right)},\sigma_{\varepsilon },\eta_{G}^{\left(s\right)},\sigma_{G}\right)$ is the agent's posterior belief about the "best" policy. Here, $\mu_{0}^{\left(o\right)}$ and $\Sigma_{0}^{\left(o\right)}$ are the ensuing first two moments of the agent's prior belief on the Other's trait. Note that, in reference to the above BPL model, the introduction of $\mu_{0}^{\left(o\right)}$ and $\Sigma_{0}^{\left(o\right)}$ is essentially an abuse of notation, since they do not include the behavioural temperature. Solving for Eq 8 yields (see S1 Text for details):

$$\left\{ \begin{array}{l} \mu_{0}^{\left(o\right)}=\frac{\sigma_{G}\;\alpha^{\left(s\right)}+\sigma_{\varepsilon }^{}\;\Gamma_{G}}{\sigma_{G}+\sigma_{\varepsilon }^{}}\\ \Sigma_{0}^{\left(o\right)}=\frac{\sigma_{G}{}^{2}\sigma_{\varepsilon }}{{\left(\sigma_{\varepsilon }+\sigma_{G}\right)}^{2}}\left(1+\frac{\sigma_{G}}{\sigma_{G}+\sigma_{\varepsilon }^{}}+\frac{\sigma_{\varepsilon }^{}\Omega_{G}}{\sigma_{G}{}^{2}}\right) \end{array}\right.$$ (9)

In brief, Eq 9 essentially states that the agent will exhibit a "false-consensus bias" [32,33], since the agent's prior $\mu_{0}^{\left(o\right)}$ on the Other's trait is a simple affine transformation of the agent's own attitude *α*^(*s*)^. Formally speaking, one could define the false-consensus bias *FCB* as the agent's prior probability that the Other's trait is identical to her own, i.e.:

$$FCB=p\left(\alpha^{\left(o\right)}=\alpha^{\left(s\right)}|\alpha^{\left(s\right)},\sigma_{\varepsilon },\sigma_{G},\Gamma_{G},\Omega_{G}\right)=\exp\left(-{\left(\alpha^{\left(s\right)}-\mu_{0}^{\left(o\right)}\right)}^{2}/2\Sigma_{0}^{\left(o\right)}\right)/\sqrt{2\pi \Sigma_{0}^{\left(o\right)}}$$ (10)

**Fig 2:**
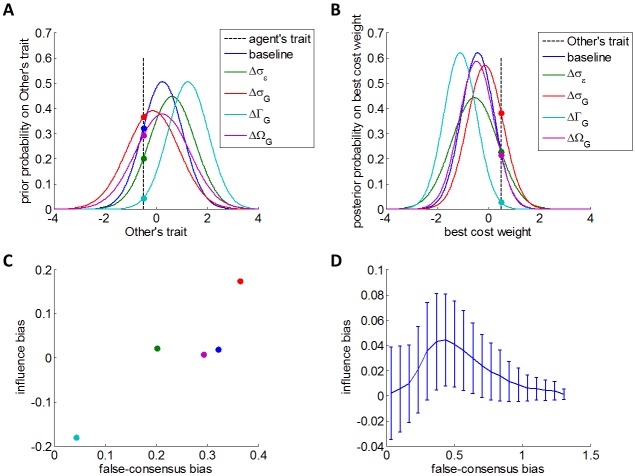
Qualitative predictions of the Bayesian model of attitude alignment. We simulated a virtual population (endowed with arbitrary cost-susceptibilities), who learn about agents performing cost-benefit arbitrages (also endowed with arbitrary cost-susceptibilities). In panels A to C, the blue colour correspond to a baseline simulation, where all model parameters have been set to unity. Other colours correspond to simulations in which each model parameter has been increased by a unitary amount in turn (*σ*_*ε*_: green, *σ*_*G*_: red, Γ_*G*_: cyan, and Ω_*G*_: magenta). The impact of model parameters can thus be eyeballed in terms of the induced changes w.r.t. the baseline simulation. **A:** false consensus bias *FCB*, as defined in Eq 10. The agent's prior probability about the Other's trait (y-axis) is plotted against possible Other's traits. The black dotted line depicts the agent's own trait, and the height at which it crosses the prior probability densities signals the false-consensus bias. **B:** influence bias *IB*, as defined in Eq 13. The agent's updated posterior belief about the "best" cost weight (y-axis) is plotted against possible "best" cost weights. The black dotted line depicts the Other's trait, and the height at which it crosses the updated posterior belief signals the influence-consensus bias (plus a correction term that derives from the agent's initial belief). **C**: influence bias *IB* (y-axis) is plotted as a function of false-consensus bias *FCB* (x-axis). This graph was constructed from the same simulations as in panels A and B. **D**: same as in panel C, but this time *IB* is averaged across 10^5^ Monte-Carlo simulations that vary randomly all model parameters (namely: *σ*_*ε*_, *σ*_*G*_, Γ_*G*_, Ω_*G*_, $\alpha_{1}^{\left(s\right)}$ and *α*^(*o*)^). The ensuing biases have been binned according to *FCB*, and error bars depict the resulting Monte-Carlo mean and standard deviation.

This definition is exemplified on Fig 2A below. One can see that the magnitude of the false-consensus bias increases with the slope of the affine transformation in Eq 9. One can show that *FCB* is maximal at the limit when agents are endowed with very vague priors (i.e. *σ*_*G*_ ≫ *σ*_*ε*_), where Eq 9 reduces to:

$$\left\{ \begin{array}{l} \mu_{0}^{\left(o\right)}\overset{\sigma_{G}\gg \sigma_{\varepsilon }}{\to }\alpha^{\left(s\right)}\\ \Sigma_{0}^{\left(o\right)}\overset{\sigma_{G}\gg \sigma_{\varepsilon }}{\to }2\sigma_{\varepsilon } \end{array}\right.$$ (11)

In brief, Eqs 9 and 10 essentially state that the false-consensus bias is indeed Bayes-optimal, given the scattering of information about "best" policies across individuals. Incidentally, they also imply that the accuracy of prior predictions regarding the Other's lazy, impatient or prudent behaviour increases with the similarity between the agent and the Other.

Importantly, observing the Other's choices informs the agent both about the Other's cost-susceptibility (cf. BPL model above) and, eventually, about the "best" policy *η*. Note that the agent's Bayesian belief update is not entirely straightforward here, because the agent has to recover the likelihood component from the Other's posterior belief about the "best" policy. Let $\alpha_{1}^{\left(s\right)}$ (resp., $\alpha_{2}^{\left(s\right)}$) be the agent's posterior mean on the "best" policy *η* before (resp., after) having observed the Other's behaviour. The agent's updated belief about the "best" policy (after having observed the Other's behaviour) can be derived as follows:

$$\begin{array}{l} p\left(\eta |a_{\to t},y^{\left(s\right)},\ldots \right)=\int p\left(\eta |a_{\to t},y^{\left(s\right)},\alpha^{o},\ldots \right)p\left(\alpha^{\left(o\right)}|a_{\to t},y^{\left(s\right)},\ldots \right)d\alpha^{\left(o\right)}\\ \;=\int p\left(\eta |y^{\left(s\right)},\alpha^{\left(o\right)},\ldots \right)p\left(\alpha^{\left(o\right)}|a_{\to t},\ldots \right)d\alpha^{\left(o\right)}\\ \;=\int \frac{p\left(\eta |y^{\left(s\right)},\ldots \right)p\left(\alpha^{\left(o\right)}|\eta ,\ldots \right)}{\int p\left(\eta |y^{\left(s\right)},\ldots \right)p\left(\alpha^{\left(o\right)}|\eta ,\ldots \right)d\eta }p\left(\alpha^{\left(o\right)}|a_{\to t},\ldots \right)d\alpha^{\left(o\right)}\\ \;≜N\left(\alpha_{2}^{\left(s\right)},\sigma_{2}^{}\right) \end{array}$$ (12)

where $p\left(\eta |y^{\left(s\right)},\ldots \right)≜N\left(\alpha_{1}^{\left(s\right)},\sigma_{1}^{}\right)$ is the agent's initial belief about the "best" policy (before having observed the Other's behaviour), *p*(*α*^(*o*)^|*η*,…) derives from Eq 7 (it describes how the Other's trait relates to the "best" policy) and *p*(*α*^(*o*)^|*a*_→*t*_,…) is the agent's posterior belief about the Other's cost-susceptibility (derived from the BPL model). Intuitively, there is an "influence bias" if the agent's updated belief about the "best" policy has drifted towards the Other's trait *α*^(*o*)^. More formally, one can define the influence bias *IB* as the increase in the agent's posterior belief that the "best" policy is identical to the Other's trait, i.e.:

$$\begin{array}{l} IB=p\left(\eta =\alpha^{\left(o\right)}|a_{\to t},y^{\left(s\right)},\ldots \right)-p\left(\eta =\alpha^{\left(o\right)}|y^{\left(s\right)},\ldots \right)\\ \;=\exp\left(-{\left(\alpha^{\left(o\right)}-\alpha_{2}^{\left(s\right)}\right)}^{2}/2\sigma_{2}^{\left(s\right)}\right)/\sqrt{2\pi \sigma_{2}^{\left(s\right)}}-\exp\left(-{\left(\alpha^{\left(o\right)}-\alpha_{1}^{\left(s\right)}\right)}^{2}/2\sigma_{1}^{\left(s\right)}\right)/\sqrt{2\pi \sigma_{1}^{\left(s\right)}} \end{array}$$ (13)

This definition is exemplified on Fig 2B above. Solving for Eq 12, one can show (see S1 Text) that the change in the agent's trait induced by observing the Other's behaviour is given by:

$$\alpha_{2}^{\left(s\right)}-\alpha_{1}^{\left(s\right)}=\lambda \left(\mu_{t}^{\left(o\right)}-\mu_{0}^{\left(o\right)}\right)$$ (14)

where $\mu_{t}^{\left(o\right)}$ is the agent's posterior mean regarding the Other's trait (after having observed *t* behavioural choices) and the "learning rate" *λ* depends upon the agent's prior precisions:

$$\lambda =\frac{1}{\frac{\sigma_{\varepsilon }^{}\Omega_{G}}{\sigma_{G}^{2}}+\frac{\sigma_{G}}{\sigma_{G}+\sigma_{\varepsilon }}+1}\Rightarrow 0\leq \lambda \leq 1$$ (15)

Eq 14 states that the agent's cost-susceptibility drifts in proportion to $\mu_{t}^{\left(o\right)}-\mu_{0}^{\left(o\right)}$, which is essentially the agent's estimate of the information that the Other holds about "best" policies (corrected for expected prior biases). Here again, Eq 14 greatly simplifies at the vague priors limit (i.e. when the false-consensus is maximal):

$$\mu_{0}^{\left(o\right)}\overset{\sigma_{G}\gg \sigma_{\varepsilon }}{\to }\alpha_{1}^{\left(s\right)}\Rightarrow \alpha_{2}^{\left(s\right)}-\alpha_{1}^{\left(s\right)}\approx \lambda \left(\mu_{t}^{\left(o\right)}-\alpha_{1}^{\left(s\right)}\right)$$ (16)

Would the agent's estimate of the Other's trait be accurate (i.e. $\mu_{t}^{\left(o\right)}\approx \alpha_{}^{\left(o\right)}$), then Eq 16 would predict a positive influence bias, whereby agents update their estimate of the "best" policy in proportion to a "prediction error" signal $\mu_{t}^{\left(o\right)}-\alpha_{1}^{\left(s\right)}$, which measures how different they are from others. In this case, the magnitude of the influence bias increases with *λ*. In particular, the maximal learning rate (*λ* ≈ 1) is achieved when *σ*_*G*_ ≫ Ω_*G*_. However, this is also when the prior variance $\Sigma_{0}^{\left(o\right)}$ is minimal, which enforces a strong shrinkage (towards $\alpha_{1}^{\left(s\right)}$) onto the agent's estimate $\mu_{t}^{\left(o\right)}$ of the Other's trait. In other terms, the vague prior limit also implies that the "prediction error" $\mu_{t}^{\left(o\right)}-\alpha_{1}^{\left(s\right)}$ will be small. In addition, away from the vague prior limit, the prior variance $\Sigma_{0}^{\left(o\right)}$ is relaxed and the agent's estimate of the Other's trait is eventually more accurate. However, the difference between $\mu_{0}^{\left(o\right)}$ and $\alpha_{1}^{\left(s\right)}$ does not cancel out and the ensuing influence bias is reduced (the agent's trait is deviated away from *α*^(*o*)^). Taken together, these remarks suggest that there may be an entangled relationship between false-consensus and influence biases. Below, we will disclose this interaction using numerical simulations.

Nevertheless, Eqs 14–16 state that the influence bias is Bayes-optimal, given that others may hold private information about "best" policies. In addition, they imply that people's change in attitude decreases with the similarity between the agent and the Other. This can be directly tested against empirical data.

In summary, Eqs 9 and 12 predict that agents observing others' lazy, impatient or prudent behaviour will exhibit false-consensus and influence biases on the respective cost-benefit arbitrages. Both these biases (i) derive from optimality principles, and (ii) are controlled by (subjective) prior assumptions regarding the scattering of information about the "best" policy across individuals. This triggers the question: is there a systematic relationship between false-consensus and influence biases, that holds when varying the four model parameters (namely: *σ*_*ε*_, *σ*_*G*_, Γ_*G*_ and Ω_*G*_) and the initial behavioural traits (namely: *α*^(*o*)^ and $\alpha_{1}^{\left(s\right)}$)? We thus conducted a series of Monte-Carlo simulations, where we sampled randomly *σ*_*ε*_, *σ*_*G*_, Γ_*G*_, Ω_*G*_, *α*^(*o*)^ and $\alpha_{1}^{\left(s\right)}$, and evaluated both false-consensus and influence biases according to Eqs 10 and 13, respectively. The results of these simulations are summarized on Fig 2 below.

Panel A (resp., B) shows how *FCB* (resp., *IB*) varies according to the model parameters, for a given set of model parameters. In this simulation, both *FCB* and *IB* increase with *σ*_*G*_, and decreases with *σ*_*ε*_, Γ_*G*_ and Ω_*G*_. The ensuing relationship between *FCB* (x-axis) and *IB* (y-axis) is shown on panel C. Panel D generalizes this analysis by summarizing the net relationship that holds across all Monte-Carlo simulations. One can see that for small false-consensus biases, *IB* increases with *FCB* (as in panel C). However, this relationship changes for higher false-consensus biases. In turn, *IB* behaves as an inverted U-shaped function of *FCB*. This partly results from the fact that *IB* eventually decreases when the mismatch between the agent's trait and the Other's is too high (cf. Fig A1 in S1 Text). In the next section, we introduce our experimental design, which relies upon a direct manipulation of this difference.

### 2. Experimental setting

#### 2.1. Participants

Fifty six healthy participants (36 females, age = 25.0 +/- 4.5 years) were recruited from the RISC network (http://www.risc.cnrs.fr/).

Data from three participants in the effort discounting tasks (see below) were excluded due to technical issues with the grip device, deception in the calibration phase or difficulty to produce any effort. We also excluded two more participants that always chose the same high-cost option in both Effort Decision phases. Moreover, in the Effort decision task, trials in which participants failed to produce the required effort were excluded from the analysis (0.2% of the Effort trials).

#### 2.2. Experimental design

**Fig 3:**
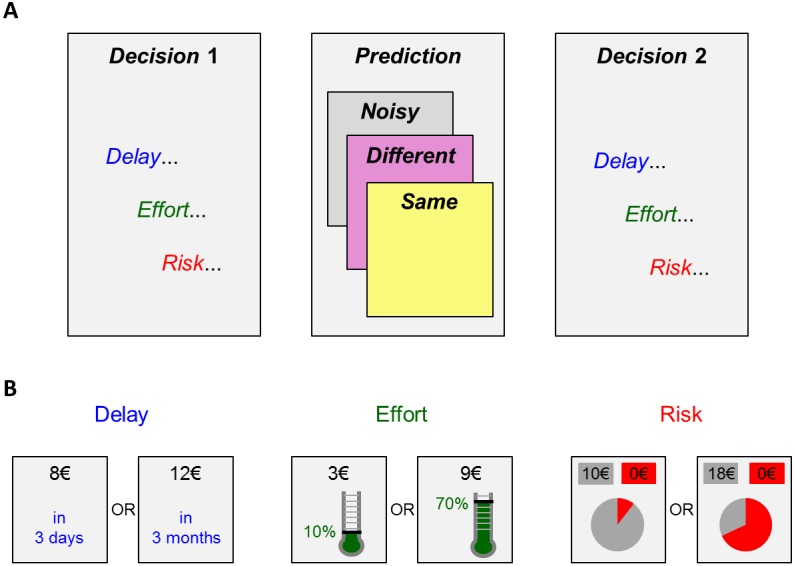
Experimental paradigm. **A:** main structure of the task (*Decision 1*, *Prediction* and *Decision 2* phases). Each phase includes three subtasks, which relate to three cost-benefit arbitrages (with three cost types, namely: delay, effort or risk, respectively). Participants are partitioned into three subgroups, depending on which cost type is paired with the *Noisy*, *Same* or *Different* condition in the *Prediction* phase. **B:** Example trials of cost-benefit arbitrages (Left: Delay, Middle: Effort, Right: Risk). Note: the low-cost/low-reward option is displayed on the left (its associated cost is fixed across trials).

In brief, our experimental design is inspired from behavioural economics choice paradigms that aim at revealing people’s attitude towards delay, effort and risk. These consist of sequences of two-alternative forced choices between two items that differ in terms of reward and cost. Here, people are requested to first make a series of such choices (*Decision* phase 1), then guess someone else's choices (*Prediction* phase), and finally re-perform the choice task (*Decision* phase 2). Critically, during the *Prediction* phase, participants are unknowingly observing choices of artificial agents (instead of human individuals), which are endowed with calibrated attitudes towards delay, effort and risk. This manipulation allows us to quantify the false-consensus and influence biases, and validate the predictions of our model. Our experimental design is summarized on Fig 3.

Each experimental phase (*Decision* 1, *Prediction* and *Decision* 2) was further subdivided into three blocks, each corresponding to a type of cost, namely: delay, effort and risk (order counterbalanced between participants). *Decision* phases were used to estimate each participant’s cost susceptibility (either delay, effort or risk) by fitting a (cost-dependant) parameterized utility function to his observed choices (cf. Eqs 1–4). Subjects were rewarded in proportion to their performance in the *Prediction* phase. In addition, one trial per *Decision* phase was randomly drawn at the end of the experiment to determine participants’ final payoff (see below).

Each block of *Decision* phases consisted of 40 trials in which participants were asked to choose between two options, i.e. a small-payoff/small-cost option (small payoffs ranged from 2€ to 12€) and a high-payoff/high-cost option (the upper bound of high payoffs was 35€). Exemplar choice alternatives are depicted on Fig 3B. To minimize cognitive load, the cost of the small payoff option was held fixed across trials (short delay, small effort, small probability of winning nothing) while the other three dimensions varied from trial to trial:

- In the delay discounting task, participants had to choose between a small payoff offered in 3 days (fixed delay) and a higher payoff later in time (delay as long as 1 year).
- For the effort discounting task, a calibration was first performed at the beginning of the experiment to measure each participant's maximal force (physiological limit) on a pneumatic grip device [50]. Participants were then asked to choose between a low-payoff/low-effort option (fixed at 10% of maximal force) and high-payoff/high-effort (until 90% of maximal force) option. After their decision was made, participants had to execute the corresponding effort on the grip device.
- In the lottery task, participants were asked to choose between a secure lottery (90% of winning a small payoff) and a riskier lottery (lower probability to get a higher payoff). The lottery outcome was not revealed during the task.

In *Decision* phase 1, the small payoff associated with the fixed cost was drawn pseudo-randomly, whereas the payoff and cost of the second option were determined by an online (trial-by-trial) adaptive design optimization procedure aiming at maximizing the efficiency of utility estimation [46,51]. We refer the reader to the S1 Text for details related to the adaptive design. In *Decision* phase 2, the same choices were presented to the participant in the same order. This ensured that changes in participants' behaviour between *Decision* phases 1 and 2 could not be due to differences in proposed alternatives. Note that this is a very conservative strategy, since people may remember and repeat their choices from *Decision* phase 1, thus potentially masking any covert influence bias.

In the *Prediction* phase, participants were asked to predict the choices from a “previous participant” (hereafter: the “Other”, in fact an artificial agent) faced with similar two-alternative forced choice tasks. On each trial, participants were first presented with the two alternative options, then asked to pick a guess, and finally informed about the Other's decision. Critically, there were three conditions (cf. Fig 2A), which corresponded to different levels of similarity between participants and artificial agents. In the *Same* condition, the Other's cost-susceptibility (*α*^(*o*)^) was chosen to be identical to the participant's cost-susceptibility ($\alpha_{1}^{\left(s\right)}$, estimated during the *Decision* 1 phase). In the *Different* condition, *α*^(*o*)^ was chosen to be different from $\alpha_{1}^{\left(s\right)}$. More precisely, we set the Other's cost-susceptibility *α*^(*o*)^ to be greater (resp., smaller) than $\alpha_{1}^{\left(s\right)}$ if $\alpha_{1}^{\left(s\right)}$ was found to be smaller (resp. greater) than the median cost-susceptibility of a pre-tested (pilot) group of participants. In these two first conditions no noise was added to the Other’s decision (null behavioural temperature, i.e.: *β*^(*o*)^ → ∞). In the *Noisy* condition, *α*^(*o*)^ was chosen identical to *α*^(*s*)^, but noise was added to the Other's decision process (the artificial agent's temperature was set to mimic the behavioural noise of the pilot participants).

To prevent carry-over effects between conditions, we used a between-subject design, with three subgroups, each of which serves as a treatment group (condition *Different*) for a given type of cost and as a control group (conditions *Same* and *Noisy*) for the other cost types. More precisely, we partitioned our participants as follows: group 1: *Different* delay discounting, *Same* effort discounting and *Noisy* risk discounting, group 2: *Noisy* delay discounting, *Different* effort discounting and *Same* risk discounting, group 3: *Same* delay discounting, *Noisy* effort discounting and *Different* risk discounting. To avoid any overlap between options presented to the participant in the *Decision* and in the *Prediction* phases, the fixed dimension in the prediction phase was different from the decision phase: the short delay was fixed to two days instead of three, the small effort 15% instead of 10% and the secure probability 85% instead of 90%. This ensured that estimates of false-consensus and influence biases were not confounded by simple anchoring or availability effects on remembered overt behaviour. In addition, another online design optimization procedure was used to determine the payoff and the cost of the second option proposed to the Other, under the constraint that the number of the Other's "impulsive" choices (low-reward/low-cost choices) was fixed (50% in each condition). This ensured that changes in participants' behaviour between *Decision* phases 1 and 2 could not be due to differences in the descriptive statistics of the Others' overt behaviour between the three conditions. In particular, would participants’ attitude change be higher in the *Different* than in the *Same* condition, then it cannot be due to a mere copying of overt behaviour.

## Results

All statistical data analyses (including classical ANOVAs and regressions) were performed using the VBA freeware (http://mbb-team.github.io/VBA-toolbox/), [46,52]. Here, we proceed with analyses of increasing sophistication. We start with model-free analyses, which rely upon simple descriptive statistics of peoples’ aggregate behaviour in both *Decision* and *Prediction* phases. We then perform another series of statistical data analyses, in the aim of directly evaluating the qualitative predictions of our computational model. These analyses exploit inter-individual differences to provide an empirical reproduction of Fig 2. Finally, we report the results of Bayesian model comparisons, given trial-by-trial choice sequences in all experimental phases.

### 1. Model-free analyses

The aim of these analyses is twofold: (i) testing for the existence of both false-consensus and influence biases on effort, delay and risk attitudes, and (ii) documenting their amplitude based on summary features of participants' data.

**Fig 4:**
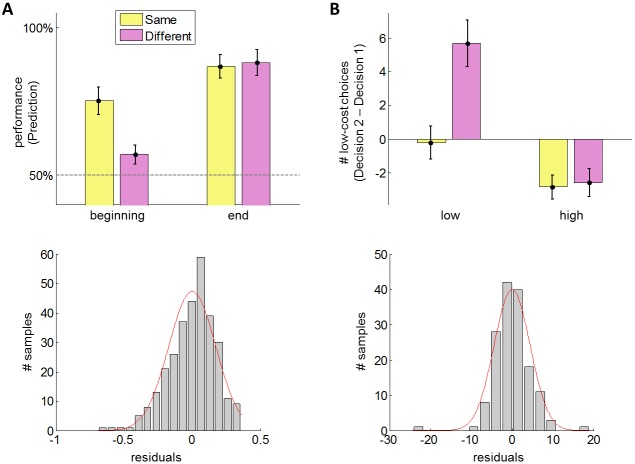
Model-free results. Quantification of false-consensus (A) and influence biases (B), in terms of a comparison between the *Same* (yellow) and *Different* (purple) conditions. **A: Top:** average performance (+/- standard error on the mean) during the *Prediction* phase is plotted as a function of session stage (beginning/end) and condition type (*Same/Different*). **Bottom:** histogram of the ANOVA residuals (grey bars) and moment-matched Gaussian approximation (red line). **B: Top:** average difference in the number of low-cost choices between *Decision* phases 1 and 2 (+/- standard error on the mean) is plotted as a function of participants’ initial cost-susceptibility (low/high) and condition type (*Same/Different*). Note: in the *Different* condition, participants with an initial high (resp., low) cost-susceptibility have observed an artificial agent endowed with a high (resp., low) cost-susceptibility. **Bottom:** histogram of the ANOVA residuals (grey bars) and moment-matched Gaussian approximation (red line).

Let us first consider the false-consensus bias, which we expect to be expressed during the *Prediction* phase, before the agent has learned about the Other's behavioural trait. Recall that, by design, the *Same* condition matches the Other's cost-susceptibility with the participant's. Thus, our prediction is twofold: (i) at the beginning of the *Prediction* phase, participants' performance should be higher in the *Same* than in the *Different* condition, and (ii) at the end of the *Prediction* phase, there should be no performance difference. We test this prediction with a pooled-variance ANOVA, where the dependant variable is the performance, which is measured in terms of percentage of correct responses (5 first/last trials in the *Same* and *Different* conditions). More precisely, we included the effects of session stage (beginning/end), condition type (*Same*/*Different*) and their interaction, as well as the effect of cost type (effort/delay/risk). Fig 4A below summarizes the performance pattern (averaged across cost types).

The ANOVA results confirm the two above predictions: there is a significant interaction between session stage and condition type (two-tailed F-test, F_1,148_ = 13.5, p = 2×10^−4^), which is due to a significant difference between the conditions *Same* and *Different* at the beginning of the session (one-tailed t-test, t_148_ = 4.68, p = 10^−5^), whereas no such difference exists at the end of the session (one-tailed t-test, t_148_ = -0.6, p = 0.70). Note that we checked ANOVA's distributional assumptions. In particular, the empirical distribution of ANOVA residuals does not significantly deviate from normality (Kolmogorov-Smirnov test: p = 0.15).

Finally, we performed a sanity check using the *Noisy* condition, which serves as a reference point for irreducible prediction errors. In particular, we asked whether the false-consensus bias induces stronger performance impairments than realistic volatility in the Other's behaviour. Post-hoc tests show that (i) at the beginning of the session, performance is significantly better in the *Noisy* than in the *Different* condition (two-tailed F-test, F_1,148_ = 21.0, p = 6×10^−6^), and (ii) at the end of the session, performance is significantly better in the *Different* than in the *Noisy* condition (two-tailed F-test, F_1,148_ = 8.0, p = 6×10^−5^). This is expected under the BPL model, since learning will be slowed down by residual prediction errors induced by noisy choices.

Regarding the influence bias, we expect that, in the *Different* condition, participants increase (resp., decrease) their cost-susceptibility if they observe somebody more (resp., less) susceptible to costs than themselves. Recall that participants face the exact same alternatives during the *Decision* phases 1 and 2. This implies that differences between the number of, e.g., low-reward choices in Decision phases 1 and 2 cannot be driven by dissimilarities between choice alternatives. However, such differences could be confounded by fluctuations in participants' cost-susceptibility around the group mean. Thus, here again, the *Same* condition serves as a control condition, this time for evaluating non-specific "regression to the mean" effects. Thus, we evaluate the experimental evidence in favour of the existence of an influence bias using a pooled-variance ANOVA, where the dependant variable is the difference in the number of low-cost choices between *Decision* phases 1 and 2, which serves as a proxy for a change in people's covert cost-susceptibility. More precisely, we included the effects of participants' initial cost-susceptibility (high/low, based upon the median-split), condition type (*Same*/*Different*) and their interaction, as well as the effect of cost type (effort/delay/risk). Fig 4B summarizes the pattern of changes in cost-susceptibility (averaged across cost types).

First of all, one can see that people tend to increase (resp. decrease) the number of low-cost choices they make when their initial cost-susceptibility is low (resp. high). Importantly, this difference is stronger in the *Different* than in the *Same* condition. The ANOVA confirms that there is a significant interaction between participants' initial cost-susceptibility and condition type (two-tailed F-test, F_1,97_ = 4.7, p = 0.03), which is due to a significant difference between the low and high initial cost-susceptibility in the *Different* condition (one-tailed t-test, t_97_ = 3.6, p = 2×10^−4^) whereas no such difference exists in the *Same* condition (one-tailed t-test, t_97_ = 1.1, p = 0.14). Post-hoc inspection of the *Different* condition shows that make people make significantly more low-cost choices in *Decision* phase 1 than in phase 2 when their initial cost-susceptibility is low, i.e. when they observed an agent more susceptible to costs than themselves (one-tailed t-test, t_97_ = 4.0, p = 6×10^−5^). Reciprocally, they make significantly less low-cost choices in *Decision* phase 1 than in phase 2 when they observed an agent less susceptible to costs than themselves (one-tailed t-test, t_97_ = 2.0, p = 0.02). Recall that these effects cannot trivially be due to simple imitation of overt choices since, by design, people observed the exact same proportion of low-cost choices in all conditions (50% for each participant and each dimension). Note however that this pattern is not perfectly in line with theoretical predictions, since people with initially high cost-susceptibility tend to decrease the number of low-cost choices in the *Same* condition. In turn, these people show no significant difference between the *Same* and the *Different* conditions. We comment on this in the Discussion section of this manuscript. Here as well, the empirical distribution of ANOVA residuals does not significantly deviate from normality (Kolmogorov-Smirnov test: p = 0.30).

Taken together, these results indicate that people become significantly more (resp., less) impatient, lazy or prudent after having observed somebody more (resp., less) susceptible to delay, effort or risk than themselves.

### 2. Validation of qualitative model predictions

So far, we have only looked at qualitative differences between the *Same* and *Different* conditions in order to assess the existence of the false-consensus and influence biases (irrespective of cost type). In what follows, we aim at evaluating the predicted relationships between one's own attitude and one's prior on the Other's trait (false-consensus), and between one's prediction error and one's attitude change (social influence), for each type of cost, separately. We assess these relationships through the analysis of inter-individual variability in prior/posterior guesses (*Prediction* phase) and initial/final cost-susceptibilities (*Decision* phases). We estimate the former by fitting the BPL model (cf. Section 1.2) to trial-by-trial data in the *Prediction* phase for each participant, in all (*Same*, *Different* and *Noisy*) conditions. We estimate the latter by fitting the cost-benefit arbitrage models (cf. section 1.1) to trial-by-trial data in the *Decision* phases for each participant, for each type of cost. Note that none of these models (BPL and cost-benefit arbitrage) are informed about either false-consensus or influence biases.

**Fig 5:**
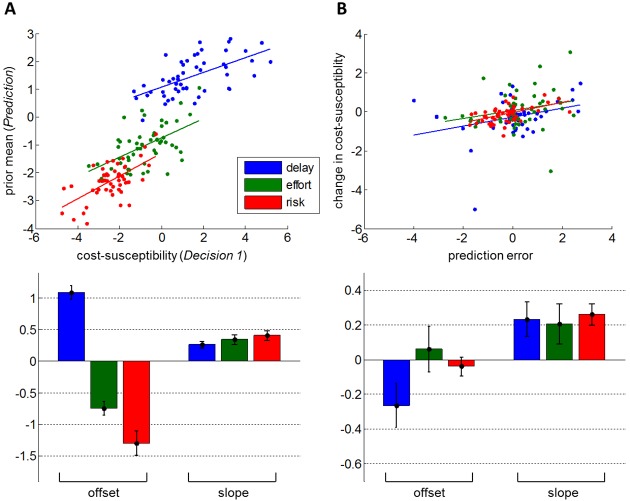
Validation of qualitative model predictions. Assessment of false-consensus (A) and influence biases (B), in terms of estimated parameters of BPL and cost-benefit arbitrage models. **A: Top**: participants’ prior mean on the Other’s cost-susceptibility (y-axis) is plotted as a function of their own cost-susceptibility (x-axis), for the three different types of cost (blue: delay, green: effort, red: risk). **Bottom**: group-level estimates (+/- standard error on the mean) of offsets and slopes for each cost type. **A: Top**: participants’ change in cost -susceptibility (y-axis) is plotted as a function of prediction error (x-axis), for the three different types of cost. **Bottom**: group-level estimates (+/- standard error on the mean) of offsets and slopes for each cost type.

Let us first consider the false-consensus bias. Recall that the BPL model is simply parameterized in terms of the mean $\mu_{0}^{\left(o\right)}$ and variance $\Sigma_{0}^{\left(o\right)}$ of the agent's prior belief $p\left(\alpha^{\left(o\right)}\right)=N\left(\mu_{0}^{\left(o\right)},\Sigma_{0}^{\left(o\right)}\right)$ on the Other's trait. We expect participants' prior $\mu_{0}^{\left(o\right)}$ on the Other's trait to behave as an affine function of their initial cost-susceptibility $\alpha_{1}^{\left(s\right)}$ (cf. Eq 9 and Fig 2A). We check this prediction by testing for a positive linear relationship between the estimated participants’ prior mean and the estimated participants’ cost-susceptibility, for each cost type. The results of this analysis are summarized on Fig 5A.

To begin with, we checked that the fitted models yield accurate accounts of participants' trial-by-trial behaviour. We thus measured the models' balanced accuracy, which is an unbiased estimate of the rate of correct predictions for binary data [53]. On average (across participants), the balanced accuracy of the cost-benefit arbitrage models (evaluated at *Decision* phase 1) was 80%, 77% and 78% for delay, effort and risk, respectively. Similarly, the average balanced accuracy of the BPL model (evaluated at the *Prediction* phase) was 88%, 85% and 79% for delay, effort and risk, respectively. Since these models achieve a high prediction accuracy, we can safely interpret the ensuing regression results. In addition, we checked whether inter-individual differences in the absolute error between final BPL posterior estimates of Other's cost-susceptibilities $\mu_{T}^{\left(o\right)}$ and true Other's cost-susceptibilities *α*^(*o*)^ predicted between-subject variability in performance during the last 5 trials of the *Prediction* phase. We found that, even when controlling for condition type, this was indeed the case for Effort (R^2^ = 12.5%, p = 0.007) and Risk (R^2^ = 11.7%, p = 0.009), but not for Delay (R^2^ = 0.2%, p = 0.37) because the end performances saturate at ceiling level (90% on average across all condition types, 93% if one discards the *Noisy* condition). Note that both cost-benefit arbitrage and BPL models were favoured by formal Bayesian model comparisons (over alternative decision and learning models). We refer the interested reader to S1 Text for details regarding statistical comparisons with other models.

We found that participants' prior mean was significantly (positively) correlated with their initial cost-susceptibility for all types of cost (one-tailed t-tests; delay: R^2^ = 36.0%, t_49_ = 5.3, p = 2×10^−6^; effort: R^2^ = 29.2%, t_49_ = 4.5, p = 2×10^−5^; risk: R^2^ = 37.8%, t_49_ = 5.5, p = 8×10^−7^). This confirms the prediction of our model, i.e. in the absence of any observation, people's prior guess about others' cost-benefit arbitrage is derived from their own cost-susceptibility.

Let us now consider the influence bias (cf. Eq 12 and Fig 2B). Our theoretical prediction is that the change in people's cost-susceptibility $\alpha_{2}^{\left(s\right)}-\alpha_{1}^{\left(s\right)}$ increases with the "prediction error" $\mu_{T}^{\left(o\right)}-\alpha_{1}^{\left(s\right)}$, where $\mu_{T}^{\left(o\right)}$ is the participant's estimate of the Other's trait (after *T* = 40 trials of the *Prediction* phase). We check this prediction by testing for a positive linear relationship between the estimated change in people's cost-susceptibility and the estimated prediction error, for each cost type. The results of this analysis are summarized on Fig 5B.

First, note that the average balanced accuracy of the cost-benefit arbitrage models (evaluated at *Decision* phase 2) was 81%, 83% and 80% for delay, effort and risk, respectively. In other terms, there is no noticeable difference in the explanatory power of the models between the *Decision* phases 1 and 2. We found that participants' change in cost-susceptibility was significantly (positively) correlated with prediction error, for all types of cost (one-tailed t-tests; delay: R^2^ = 10.1%, t_49_ = 2.3, p = 0.01; effort: R^2^ = 6.0%, t_49_ = 1.8, p = 0.04; risk: R^2^ = 26.9%, t_49_ = 4.2, p = 5×10^−5^). Although directly validating Eq 16, a positive correlation of this sort could be partly confounded by the common term in both dependent and independent variables (namely: $\alpha_{1}^{\left(s\right)}$). It turns out that one can simply re-arrange Eq 16 to yield: $\alpha_{2}^{\left(s\right)}\approx \lambda \;\mu_{T}^{\left(o\right)}+\left(1-\lambda \right)\alpha_{1}^{\left(s\right)}$. This suggest another (more indirect) way of validating Eq 16, by regressing $\alpha_{2}^{\left(s\right)}$ against both $\mu_{T}^{\left(o\right)}$ and $\alpha_{1}^{\left(s\right)}$. Here, evidence for an influence bias derives from the significance of the contribution of $\mu_{T}^{\left(o\right)}$. Note that $\mu_{T}^{\left(o\right)}$ and $\alpha_{1}^{\left(s\right)}$ are anti-correlated by design, which is why we orthogonalized $\alpha_{1}^{\left(s\right)}$ w.r.t. $\mu_{T}^{\left(o\right)}$ prior to performing the multiple regression analysis. Eventually, we found that $\mu_{T}^{\left(o\right)}$ explained a significant amount of variance, for all types of cost (one-tailed t-tests; delay: R^2^ = 46.9%, t_48_ = 6.5, p = 2×10^−8^; effort: R^2^ = 22.6%, t_48_ = 3.7, p = 2×10^−4^; risk: R^2^ = 71.6%, t_48_ = 11.0, p<10^−8^). Of course, -orthogonalized- $\alpha_{1}^{\left(s\right)}$ was also found to significantly contribute to inter-individual variability in $\alpha_{2}^{\left(s\right)}$ (delay: p<10^−8^; effort: p = 2×10^−7^; risk: p<10^−8^). Taken together, these analyses confirm the prediction of our model, i.e. people align their attitude towards delay, effort and risk with others'.

Finally, we tested the inverted U-shaped relationship that we expect to hold between the false-consensus and the influence biases (cf. Fig 2). Note that, in contradistinction to the false-consensus bias, we cannot directly apply the formal definition of the influence bias. This is because the derivation of *IB* in Eq 13 requires the specification of parameters from the Bayesian model of attitude alignment. However, we took inspiration from [8], and used a model-free proxy for *IB*, i.e.:

$$I\hat{B}=\frac{\alpha_{2}^{\left(s\right)}-\alpha_{1}^{\left(s\right)}}{\alpha_{}^{\left(o\right)}-\alpha_{1}^{\left(s\right)}}$$ (17)

**Fig 6:**
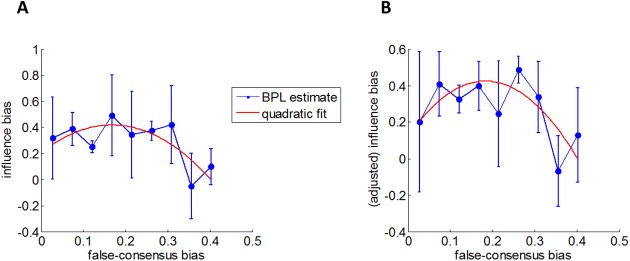
Assessment of the relationship between false-consensus and influence biases. This figure summarizes the attempt to validate the prediction summarized on Fig 2D. **A:** participants' influence bias $I\hat{B}$ (y-axis) is plotted as a function of their false-consensus bias *FCB* (x-axis), in the *Different* condition, across all cost types. The data have been binned according to *FCB*, and blue error bars depict the resulting mean and standard errors around the mean. The red line depicts the best fitting quadratic expansion. **B:** Same as in A, but this time with influence biases corrected for age and gender inter-individual variability.

Using the former numerical Monte-Carlo simulations, we verified that the correlation between *IB* and $I\hat{B}$ was indeed very high (r = 0.79), and that $I\hat{B}$ also behaved as an inverted U-shaped function of *FCB*. In particular, when fitting a quadratic model of *FCB* to *IB* and $I\hat{B}$, the quadratic regressor explained 5.3% of the variance of *IB* and 7.4% of $I\hat{B}$. We then performed this analysis with estimated behavioural traits in the *Different* condition (because $I\hat{B}$ is ill-defined when $\alpha_{}^{\left(o\right)}-\alpha_{1}^{\left(s\right)}$), having pooled the data across cost types. The latter is justified both by the predicted low statistical power of the test, and because we expect the inverted U-shaped relationship to hold across subjective attributes of attitude alignment. The results of this analysis are summarized on Fig 6 below.

First of all, we asked whether the average influence bias, as proxied with $I\hat{B}$, was significant. This was indeed the case (one-tailed t-test: t_48_ = 4.4, p = 6×10^−5^, R^2^ = 29.3%). In addition, there was no strong difference across cost types (F-test: F_2,47_ = 2.4, p = 0.1, R^2^ = 9.3%). Then, we asked whether $I\hat{B}$ follows an inverted U-shape relationship with *FCB*. When binning the data according to *FCB* (cf. panel 6.A), this relationship is barely visible, due to high between-subject variability. In fact, when fitting the quadratic model on un-binned data, the quadratic regressor showed a trend that did not reach statistical significance (one-tailed t-test: t_47_ = 1.3, p = 0.09, R^2^ = 3.6%). We could only reveal the quadratic effect above between-subject variability when including both participants' age and gender as possible confounding factors in the regression model. The resulting analysis is shown on panel 6.B. In this case, the quadratic effect was deemed significant (one-tailed t-test: t_45_ = 1.7, p = 0.04, R^2^ = 6.0%). Note that the empirical histogram of the ensuing residuals shows no significant deviation to normality (Kolmogorov-Smirnov test: p = 0.51). We will comment on these results in the Discussion section below.

### 3. Model-based analyses

In brief, the above analyses highlight the existence of false-consensus and influence biases, which verify the main predictions of our Bayesian model (according to classical significance testing procedures). However, we have not yet addressed the issue of assessing the model's explanatory power, given the full set of participants' trial-by-trial behavioural data (across all *Decision* and *Prediction* phases). In particular, under our social Bayesian model, the false-consensus and influence biases are related through people's priors regarding the scattering of information about "how good" cost-benefit arbitrages are (cf. section 3.3). In what follows, we perform a statistical comparison of our model, with and without false-consensus and/or influence biases, given trial-by-trial choice sequences in all experimental phases.

In principle, one can pool the cost-benefit arbitrage models (for *Decision* phases 1 and 2) with the BPL (for the *Prediction* phase) in four different ways, depending on whether one includes or not the false-consensus bias (according to Eq 9) and/or the influence bias (according to Eqs 14 and 15). On the one hand, the absence of a false-consensus bias can be simply accounted for by releasing the moments of the agent's prior ($\mu_{0}^{o}$ and $\Sigma_{0}^{o}$) on the Other's behavioural trait from the constraint of Eq 9. This simply means that they belong to the set of unknown model parameters, which have to be estimated given participants' behaviour. In turn, this decouples the *Prediction* phase from the first *Decision* phase (they share no common parameter). Note that this still leaves open the possibility of other sorts of prior guesses about others' behavioural trait. On the other hand, the absence of an influence bias is simply modelled by equating the agent's initial and final cost-susceptibility (in *Decision* phases 1 and 2, respectively). This effectively decouples *Decision* phase 2 from the *Prediction* phase (no influence bias).

We thus considered the following 2×2 factorial model space for each participant and each cost type (delay, effort and risk): *m*_1_ (no false-consensus bias, no influence bias), *m*_2_ (false-consensus bias, no influence bias), *m*_3_ (no false-consensus bias, influence bias) and *m*_4_ (false-consensus and influence biases). Model parameters were allowed to vary across participants and cost types. To account for model complexity when quantifying how likely these models are given the participants' choice sequences, we evaluated their marginal likelihood or Bayesian model evidence, under a variational-Laplace approximation [46,47,52]. These were then inserted into a group-level random-effect Bayesian model comparison (RFX-BMS) [54,55]. This analysis treats models as random effects that could differ between participants, with an unknown population distribution (described in terms of model frequencies/proportions). In particular, we report the exceedance probability (EP) associated with models (or family of models), which corresponds to the posterior probability that a given model is the most frequent one in the population.

**Fig 7:**
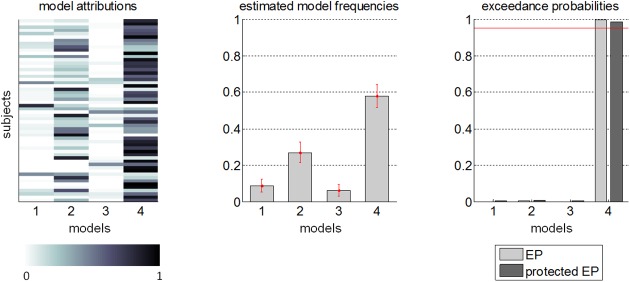
Model-based results. Evaluation of the joint treatment of false-consensus and influence biases, in terms of a group-level statistical comparison of four candidate models of participants’ behaviour over all *Decision* and *Prediction* phases (m_1_: no false-consensus, no influence; m_2_: false-consensus, no influence; m_3_: no false-consensus, influence; m_4_: false-consensus and influence). **Left:** model attributions for each model (x-axis) and each participant (y-axis). **Middle:** estimated model frequencies (+/- posterior standard error) of each model. **Right:** exceedance probabilities (light grey) and protected exceedance probabilities (dark grey) of each model.

To begin with, between-condition comparisons allows us to ask whether model attributions are stable across cost types [54]. It confirms that the underlying model (within the comparison set) is invariant across cost types (EP = 100%). Thus, we sum log-evidences over cost types (fixed effect across cost types), and performed RFX-BMS. The results of this analysis is summarized in Fig 7.

Let us first consider inter-individual differences in model attributions, i.e. the posterior probabilities that each model explains each participant's data. These are essentially summary statistics of Bayesian model comparison at the subject-level. One can see that, although a few participants seem to show no evidence of influence bias (model *m*_2_), model attributions mostly supports model *m*_4_, i.e. the presence of both false-consensus and influence biases, as predicted by Eqs 9, 14 and 15. We refer the interested reader to S1 Text for reports of best and worst model fits (across participants). This eventually transpires in the RFX-BMS estimates of model frequencies, which account for within-subject uncertainty regarding model attributions. In brief, about 85% of people are best described in terms of either model *m*_4_ (frequency ± two standard deviations = 58.1% ±6.3%) or model *m*_2_ (26.9% ±5.7%). The Bayesian omnibus risk was very low (BOR = 1.6%), indicating that this pattern of model frequencies is very unlikely driven by chance [54]. Finally, the exceedance probability shows that model *m*_4_ is likely to be most prevalent in the population (EP = 99.6%, protected EP = 98.4%).

Taken together, these analyses provide an independent piece of evidence in favour of our Bayesian interpretation of attitude alignment, which results from the interaction between false-consensus and influence biases.

## Discussion

In this paper we introduce and validate a computational model of how people learn about and from others' lazy, impatient or prudent attitudes. This model predicts that such learning exhibits both false-consensus and influence "biases", which arise from Bayes-optimal information processing. It also predict that the magnitude of attitude alignment results from a non-trivial interaction between false-consensus and influence biases. In a behavioural experiment, we show that healthy adults behave in accordance to the predictions of the model. In particular, we demonstrate a strong false-consensus bias, whereby people's prior guess about others is derived from their own attitude, and is then refined with observational information. In addition, people's cost-benefit arbitrage drifts towards others', leading to an influence bias on effort, delay and risk attitudes. Finally, the influence bias seems to follow the predicted inverted U-shaped relationship with false-consensus.

Let us first discuss how consistent and/or novel our results are with respect to the existing literature

First of all, we found that people are capable of learning about others' lazy, impatient or prudent behaviour (reaching a choice prediction accuracy of about 85%, cf. Fig 4A). Moreover, our results suggest that this learning skill relies upon sophisticated mentalizing, which is bound to a false-consensus bias that gradually disappears as informative feedbacks are provided. From an experimental viewpoint, this result is in line with recent neuroscientific studies, which confirmed that typical mentalizing brain systems -more precisely, medial prefrontal regions- are involved when people are predicting others' delay arbitrages [56] and choices in a learning task [57]. However, these studies were neither in a position to detect false-consensus biases, nor did they provide any computational insight into how people mentalize in such tasks. Our work also extends previous computational studies, which indicate that people are more likely to rely on Bayesian mentalizing schemes than on heuristics when predicting others' choices over good bundles [58]. Here, the incremental aspect of our work is twofold: we disclose a computationally efficient (variational) approximation to exact Bayesian inference (cf. S1 Text), and (ii) we incorporate both false-consensus and influence mechanisms into the mentalizing process. The former issue is extremely relevant to neuroscientific inquiries because it suggest a simple algorithmic implementation of mentalizing that could be traced back in neuroimaging trial-by-trial signals. The latter issue is important, because it sheds new light on a vast amount of empirical findings from social psychology. In particular, an established view on "false consensus effects" is that it fulfils the need for self-esteem, in that it deludes people into thinking that their values or beliefs are shared by others [59]. We refer the interested reader to [60] for variants of this type of explanations. Here, we argue that one does not need to resort to constructs of this sort. Rather we suggest that the ultimate cause of false-consensus biases is that they (paradoxically) improve mentalizing performance (in terms of behavioural prediction accuracy). We will come back to this later on, when discussing the optimality of false-consensus and influence biases.

Second, we found that people learn from others’ lazy, impatient or prudent behaviour. More precisely, we have shown that people's attitude towards effort, delay or risk drifts towards that of others. This extends previous findings regarding similar “social contagion” effects on aesthetic judgments [18,61,62]. To the best of our knowledge, only two studies discuss the influence of others' covert attitudes [8,9]. Their inconsistent results (in terms of the significance of delay discounting influence) may be due to differences in experimental designs, which may not have afforded enough statistical power to detect this effect. In this context, our results are important, because the influence bias may explain some form of preference instability, which has been largely documented in the behavioural economics literature [63–65]. A related question is whether such phenomena are driven by simple public compliance that only induces short-term changes in overt preference ratings [66] or reflect more enduring changes in deep determinants of behaviour [67]. Although we cannot directly address this question, we provide empirical evidence that the influence of others' attitude towards effort, risk or delay is not simply due to the imitation of overt behaviour. This is because, in our experimental setting, the influence bias occurs despite the fact that the statistical properties of observed overt behaviour (such as the number of low-cost choices) were kept identical across conditions. In any case, our study is the first to demonstrate the impact of computational properties of mentalizing upon attitude alignment. In particular, we have disclosed the non-trivial interaction between false-consensus and influence biases, which was predicted by the Bayesian model of attitude alignment we have proposed. Although further work is clearly needed to establish this effect and explore its practical relevance, this result is undoubtedly unprecedented.

Let us now discuss the limitations of our study

First of all, recall that we performed three types of statistical data analyses, with increasing sophistication (from model-free summary statistics to validations of qualitative model predictions, to quantitative model comparisons given trial-by-trial choice sequences). Beyond its internal consistency, one could question the strength of the reported empirical evidence. For example, using model-free analyses, we found that people with initial high-cost susceptibility changed their choices similarly in the conditions *Same* and *Different*. This is problematic because the condition *Same* serves to evaluate baseline uncontrolled preference instabilities (including, e.g., regression to the mean effects). This is however, likely imputable to a combination of weaknesses in our design, namely: the fact that we used a between-subject design (where inter-individual differences may partly confound the comparison of the *Same* and the *Different* conditions), our use of a prior pilot study for partitioning subjects in *High* versus *Low* initial cost-susceptibility subgroups (which resulted in slight statistical imbalance, whereby the variance of the prediction error $\alpha_{}^{\left(o\right)}-\alpha_{1}^{\left(s\right)}$ was higher in the *High* group than in the *Low* group), etc… All these issues may have resulted in a loss of statistical power, which would explain the absence of evidence for a difference between the *Same* and the *Different* conditions for people with initial high-cost susceptibility. One may also ask whether the empirical evidence for the false-consensus bias may not be deemed stronger than for the influence bias. A simple possibility is that the false-consensus bias may be a more stable phenomenon than the social influence bias. This is perhaps best exemplified in the evaluation of the qualitative model predictions, in terms of the effect sizes of related affine transforms (cf. Fig 4). In fact, a simple two-sample t-test confirms that the difference in percentages of explained variance is significant (p = 0.02). Recall, however, that our Bayesian model of attitude alignment was predicting that the affine relationship between the agent's prior and her initial attitude should be much less noisy than the relationship between her attitude change and the prediction error. This is perhaps best exemplified on Figure A1 of S1 Text. In relation to this, although we found evidence for an inverted U-shaped relationship between false-consensus and influence biases, we acknowledge that this evidence is not definitive. Recall that the quadratic effect only became significant when we explained away between-subject variability that was induced by inter-individual differences in age and gender. Here again, although the predicted statistical power was rather weak (expected R^2^ = 7.4%), we had not optimized the experimental design w.r.t. the detection of this effect. Clearly, further work is needed here to firmly establish this effect.

Second, despite the converging empirical evidence reported in this study, one may argue that the validation of our Bayesian model of attitude alignment is incomplete. For example, we did not include any explicit comparison with alternative explanations, which would make qualitatively similar predictions. The issue here is in identifying scenarios that can explain how people learn about and from others' lazy, impatient or prudent behaviour the way they do; in particular, these scenarios have to account for both false-consensus and influence biases. For example, during the Prediction phase, if people do not learn some form of (hidden) parameterized utility function when predicting others' cost-benefit arbitrages, what do they learn? A possibility is that people update an estimate of the probability that others' will choose the low-cost option. However, Bayesian model comparison with the BPL dismisses this assumption (see S1 Text). More generally, no simple heuristic strategy based upon the statistics of overt behaviour would work here, because these are matched across conditions (by design, the frequency of observed low-cost choices is fixed to 50%). In other terms, learning is impossible without explicitly accounting for the cost-benefit properties of choice alternatives. This is why simple variants of reinforcement learning models (as used in, e.g., [57]) cannot explain how people eventually learn to predict others’ cost-benefit arbitrages. Also, one may ask how close people's internal models of others' cost-benefit arbitrages are to the mathematical form of the specific utility functions we employed to simulate attitudes towards delay, effort or risk. In fact, our experimental claim does not go as far as to assert that behavioural economic models are best representatives of people's mentalizing intuitions. Rather, they serve as simple proxies for adjustable cost-benefit arbitrages, which can account for people's sensitivity to inter-individual differences. But then: what if our selection of ad-hoc utility functions provides distorted representations of people's mentalizing processes? This is of course theoretically possible. Nevertheless, Bayesian model comparison of different underlying utility functions (including linear utilities) in the *Prediction* phase supports the current selection of cost-specific utility functions (see S1 Text).

Third, we acknowledge that our theoretical framework falls short of related psychologically-relevant objectives. To begin with, our Bayesian model cannot be used to predict which internal aspects of mentalizing are conscious/explicit and which are unconscious/implicit. Recall that this as a general limitation of models based upon information theory, which ignores this distinction. This is because the conscious/unconscious dichotomy makes a difference neither to its mathematical constituents (probability distributions) nor to their manipulation (probability calculus). In brief, a Bayesian agent is neither conscious nor unconscious: it is automatic. This might be considered unfortunate, given the current debate regarding the relative contributions of implicit and explicit components of mentalizing [24,68–70]. Note that the analysis of the experiment's written debriefing indicates that only 19% of the participants became aware of a change in their attitude towards effort, delay or risk. Interestingly, awareness of attitude change does not correlate with its magnitude. We thus are inclined to think that, although partially accessible to some form of metacognitive self-monitoring, the influence bias is likely to be mostly implicit. This is in line with studies reporting "blindness" or social influence on categorical judgments [71,72]. Yet another limitation of our model relates to the dynamics of attitude alignment. Strictly speaking, the model specifies neither when attitude alignment occurs nor how long it lasts for. Addressing the former issue would require comparing model variants in which attitude alignment occurs either while people are mentalizing about others, or when they are about to perform cost-benefit arbitrages. A first hint here comes from recent neuroscientific evidence, which indicates that preference malleability occurs in parallel to learning-induced plasticity in medial prefrontal cortex [8]. The latter issue is related to the robustness of the influence bias. Owing to the relative weakness of the effect sizes reported in this study (at least when compared to the false-consensus bias), we would argue that further work is clearly needed here.

Let us now discuss the Bayes-optimality of false-consensus and influence biases in the light of evolutionary thinking. We have argued that these phenomena should be expected, from information-theoretic principles. More precisely, our Bayesian model of attitude alignment suggest that they arise from prior assumptions regarding the scattering of people's information about how “good” behavioural policies are. This joint treatment of false-consensus and influence biases deserves a few clarifying comments.

Recall that, beyond the context of attitude alignment, Bayesian models of cognition seem to be paradoxically plagued by their (somewhat undesirable) normative perspective on information processing [73,74]. This sort of criticism undoubtedly applies to the work we present here. We acknowledge that it may be difficult, if not impossible, to claim that the learning mechanisms behind false-consensus and influence biases are quintessentially Bayesian. In fact, it would be almost trivial to capture, e.g., the influence bias, using ad-hoc learning models (e.g., variants of Rescorla-Wagner learning rules). However, such models would not provide an explanation as to why one should expect to see an influence bias in the first place. In addition, it is unlikely that models of this sort would predict more intricate phenomena, such as the existing interaction between false-consensus and influence biases. From our point of view, here lies the value of our Bayesian account of attitude alignment: it predicts most (if not all) of the salient features of these biases from first (normative) principles.

We start with the premise that one's behavioural trait can be thought of as one's subjective and uncertain estimate of the "best" policy. It follows that one may guess others' cost-susceptibility from one's own belief about "best" policies, hence the false-consensus bias. In turn, the false-consensus bias results from the combination of two necessary factors, namely: (i) one’s behavioural trait determines one's prior expectation for others’, and (ii) the precision of this prior guess is not null (finite prior variance). Note that, in principle, the prior bias that would eventually yield the fewest prediction errors on average should be derived from the distribution of traits within the population. So why is there any false-consensus bias at all? The reason lies in the non-trivial relationship between the false-consensus and influence biases. As one aligns with more and more individuals, one’s trait would slowly converge to the group mean (up to sampling errors). In turn, the false-consensus bias would tend towards the optimal prior.

Let us now turn to the influence bias, which we see as yet another consequence of the above premise. Similarly to information cascades in serial judgments [75], being exposed to others' attitudes towards delay, effort or risk provides the observer with an opportunity to learn utile information regarding "best" policies. At this point, there are two (related) remarks to be made. First, a given environmental niche might favour some behavioural traits through selective pressure. Critically, whether attitude alignment facilitates or hinders this distal selection pressure is a nontrivial question [76]. The key idea here, is that attitude alignment may have adaptive fitness, essentially because it serves to correct potentially inaccurate (oddball) innate traits. To support this claim, one would need to show, using, e.g., Evolutionary Game Theory [77], that phenotypes exhibiting attitude alignment (as documented in this study) eventually persist, irrespective of the adaptive fitness of any particular attitude (which depends upon arbitrary features of the socio-environmental niche). Second, our treatment of attitude alignment bypasses established evolutionary explanations that rely on social norms and the avoidance of social rejection [67,78,79]. Note that under the latter view, false-consensus biases are but a form of "wishful thinking" [80], i.e. a (pleasant?) delusion of conformity. But then, wouldn't false-consensus aggravate the risk of social rejection, hence eventually impairing the adaptive fitness of social agents? This is important, because it would imply that social conformity and "oddball correction" would act as opposing evolutionary forces on phenotypes that exhibit both influence and false-consensus biases. Here again, Evolutionary Game Theory can be used to identify which features of the species' socio-environmental niche would control which of these forces would dominate.

These remarks are in fact reminiscent of the debate regarding the evolutionary origins of "herding", i.e. the uncoordinated alignment of behaviours of individuals in a group (herd) that occurs without centralized coordination [81]. Early field investigations suggested that herding can emerge from the uncoordinated behaviour of animals engaged in, e.g., predator avoidance or foraging [82]. More generally, herding behaviour includes, but is not limited to, insect swarming [83], bird flocking [84] or human crowding [85]. The latter takes impressively diverse forms, ranging from panic crowd behaviour [86,87], to fashions, cultural customs and the localized conformity of political opinions [75,88]. The breadth of the phenomenon extends largely beyond that of human attitude alignment, as can be possibly captured by the model we have presented here. Nevertheless, attitude alignment may be viewed as one of the many cognitive mechanisms that contribute to the self-organization of collective (human?) behaviour [89,90]. The non-trivial issue here is to predict the transient dynamics of social group interactions that result from the intrinsic properties of attitude alignment [91]. This unresolved question opens many fascinating questions that may require the development of novel experimental and theoretical tools [92].

## Supporting information

S1 Text Supporting information. This document provides additional information regarding models’ mathematical derivations, statistical methods, experimental details, additional data analyses and model inversion diagnostics.
(PDF)
