# Supplementary material for: Learning about and from others' prudence, impatience or laziness: The computational bases of attitude alignment
Source: PLoS Comput Biol. 2017 Mar 30;13(3):e1005422. doi: 10.1371/journal.pcbi.1005422 (PMC5373523; doi:10.1371/journal.pcbi.1005422)
Supplement: S1 Text — This document provides additional information regarding models’ mathematical derivations, statistical methods, experimental details, additional data analyses and model inversion diagnostics. (PDF) [file pcbi.1005422.s001.pdf]

Learning about and from others' prudence, impatience or laziness: the computational bases of attitude alignment [supplementary information].

M. Devaine[1], *J. Daunizeau*[1,2]

[1] Brain and Spine Institute, Paris, France

[2] ETH, Zurich, Switzerland

I. Mathematical derivation of the BPL model.

In what follows, we provide mathematical details regarding the derivation of our "Bayesian Preference Learner" or BPL model. This model essentially describes how Bayesian learners update, on a trial-by-trial basis, their estimate of the others' cost-susceptibility parameter α (as well as the inverse-temperature β) from observed choices.

Recall that mentalizing Bayesian agents assume that the *Other*'s choices obey the softmax decision rule of Equation 1 of the main text. This yields the following binomial likelihood $p\left(a_{\to t}\middle|\theta^{(o)}\right)$ for the Other's decision $a_{\to t}$ (up to trial t):

$$
\begin{aligned}
p\left(a_{\to t}\middle|\theta^{(o)}\right) &= \prod_{t'=1}^{t} p\left(a_{t'}\middle|\theta^{(o)}\right) \\
p\left(a_t\middle|\theta^{(o)}\right) &= s\left(f\left(\theta^{(o)}\right)\right)^{a_t}\left(1-s\left(f\left(\theta^{(o)}\right)\right)\right)^{a_t-1} \\
f\left(\theta^{(o)}\right) &= \beta^{(o)}\left(u_{\alpha^{(o)}}(\mathrm{Op}_1)-u_{\alpha^{(o)}}(\mathrm{Op}_2)\right)
\end{aligned}
\qquad \text{(A1)}
$$

where $s: x \to s(x) = 1/1+e^{-x}$ is the sigmoid function, $a_t \in \{0,1\}$ is the Other's binary choice at trial t, $s\left(f\left(\theta^{(o)}\right)\right) \triangleq p\left(a_t = 1\middle|\theta^{(o)}\right)$ is the probability that the Other chooses the first alternative option and $\theta^{(o)} = \left\{\log\alpha^{(o)}, \log\beta^{(o)}\right\}$ gathers both the Other's cost-susceptibility $\alpha^{(o)}$ and her inverse-

temperature $\beta^{(o)}$. Note that the log transform effectively enforces a positivity constraint on both cost-susceptibility and temperature.

Before having observed any Other's decision, the agent is endowed with some prior belief $p\left(\theta^{(o)}\right)$ about the Other's behavioural trait $\theta^{(o)}$. Without loss of generality, we assume that this prior belief $p\left(\theta^{(o)}\right)=N\left(\mu_0^{(o)},\Sigma_0^{(o)}\right)$ is Gaussian with mean $\mu_0^{(o)}$ (which captures the direction of the agent's bias) and variance $\Sigma_0^{(o)}$ (which measures how uncertain is the agent's prior belief).

Observing the Other's choices gives the agent information about $\theta^{(o)}$, which can be updated trial after trial using the following Bayes-optimal probabilistic scheme:

$$\begin{aligned} p\left(\theta^{(o)} \middle| a_{\to t}\right) &\propto p\left(a_{\to t} \middle| \theta^{(o)}\right) p\left(\theta^{(o)}\right) \\ &\propto p\left(a_t \middle| \theta^{(o)}\right) p\left(\theta^{(o)} \middle| a_{\to t-1}\right) \end{aligned} \tag{A2}$$

where $p\left(\theta^{(o)} \middle| a_{\to t}\right)$ is the agent's posterior belief about the Other's behavioural trait after trial t and the second line highlights the sequential (online) form of Bayesian belief update.

Equation A2 can be approximated using a variational-Laplace scheme, which essentially replaces the integration implicit in Equation A2 with an optimization of the sufficient statistics of the approximate posterior distributions (Daunizeau et al., 2014; Friston et al., 2007). This eventually yields semi-analytical expressions for the trial-by-trial update rules of two first moments of the posterior probability density function. In brief, we approximate the posterior belief $p\left(\theta^{(o)} \middle| a_{\to t}\right) \approx N\left(\mu_t^{(o)},\Sigma_t^{(o)}\right)$ in terms of a Gaussian distribution with mean $\mu_t^{(o)}$ and variance $\Sigma_t^{(o)}$, which are updated as follows:

$$\begin{cases} \Sigma_t^{(o)} = \left(\Sigma_{t-1}^{(o)-1} + s\left(f\left(\mu_{t-1}^{(o)}\right)\right)\left(1-s\left(f\left(\mu_{t-1}^{(o)}\right)\right)\right) \nabla f\big|_{\mu_{t-1}^{(o)}}{}^{T} \nabla f\big|_{\mu_{t-1}^{(o)}}\right)^{-1} \\ \mu_t^{(o)} = \mu_{t-1}^{(o)} + \Sigma_t^{(o)} \nabla f\big|_{\mu_{t-1}^{(o)}} \left(a_t - s\left(f\left(\mu_{t-1}^{(o)}\right)\right)\right) \end{cases} \tag{A3}$$

where $\nabla f = \partial f / \partial\theta^{(o)}$ is the gradient of the weighted value difference f (cf. Equation A1) w.r.t. the behavioural trait parameters $\theta^{(o)}$. It can be seen from Equation A3 (first line) that the agent's posterior uncertainty about the Other's behavioural trait is monotonically decreasing over trials. Moreover, the agent is accumulating information at a rate that depends upon the properties of the choice alternatives (differences in reward and cost). Also, the change in the agent's posterior mean $\mu_t^{(o)} - \mu_{t-1}^{(o)}$ is driven by a prediction error (i.e. $a_t - s\left(f\left(\mu_{t-1}^{(o)}\right)\right)$), whose impact is modulated by the agent's subjective uncertainty $\Sigma_t^{(o)}$. Note that the prediction error $a_t - s\left(f\left(\mu_{t-1}^{(o)}\right)\right)$ can be explained away either by updating the posterior estimate of the Other's cost-susceptibility, or by increasing the posterior estimate of the temperature. In turn, the impact of the next prediction error will be smaller, i.e. it will induce a smaller update $\mu_t^{(o)} - \mu_0^{(o)}$. In other words, the rate of posterior update $\mu_t^{(o)} - \mu_0^{(o)}$ decreases as the true Other's cost-susceptibility $\alpha^{(o)}$ further departs from the prior mean $\mu_0^{(o)}$. This eventually bounds the estimate of Other's cost-susceptibility $\alpha^{(o)}$. We refer the interested reader to (Devaine et al., 2014b; Mathys, et al., 2011) for further mathematical details regarding the derivations of similar meta-Bayesian learning rules.

Iterated through time or trials, Equation A3 essentially describes how the agent learns about the Other's lazy, impatient or prudent attitude. Given the Other's choices up to trial t, the agent can now form a prediction about the Other's preference at trial $t+1$:

$$\begin{aligned} E\left[a_{t+1} \middle| \mathrm{Op}_{t+1}, a_{\to t}\right] &= E\left[s\left(f\left(\theta^{(o)}\right)\right) \middle| a_{\to t}\right] \\ &\approx s\left(f\left(\mu_t^{(o)}\right)\right) \end{aligned} \tag{A4}$$

Equations A3 and A4 complete the exposition of our "Bayesian Preference Learner" or BPL model.

II. Mathematical derivation of the Bayesian model of attitude alignment.

In what follows, we provide mathematical details regarding the derivation of our Bayesian model of attitude alignment. We consider the case of cost-benefit arbitrages, whereby peoples' subjective preferences control the relative weight of rewards and costs. In brief, we suppose that peoples' subjective preferences are essentially peoples' belief regarding the "best" (domain-specific) policy. More precisely, people form a subjective estimate of the "optimal" cost-susceptibility η for each type of cost-benefit arbitrage. Such "optimal" cost-susceptibility can be thought as yielding cost-benefit arbitrages that are most adapted in the agent's environmental niche. Peoples are endowed with an innate prior regarding what η might be, which they update given two sources of information: noisy reinforcement signals and uncertain observations of others' attitudes. We will see that, under these premises, a Bayesian agent eventually expresses both false-consensus and influence biases.

Let $p\left(\eta \middle| \eta_G^{(i)}, \sigma_G\right) \triangleq N\left(\eta_G^{(i)}, \sigma_G\right)$ be the prior distribution of agent i about the best policy η, which we parameterize in terms of its agent-dependent mean $\eta_G^{(i)}$ and variance σ_G. For sake of simplicity, we assume that σ_G is the same for everyone and that the agents' prior mean $\eta_G^{(i)} \sim N\left(\Gamma_G, \Omega_G\right)$ is scattered across individuals as a Gaussian variable with mean Γ_G and variance Ω_G. The environment provides agent i with noisy feedback $y^{(i)}$ regarding the optimal policy, i.e. $y^{(i)}$ is related to η as follows: $y^{(i)} = \eta + \varepsilon^{(i)}$, where $\varepsilon^{(i)} \sim N\left(0, \sigma_\varepsilon\right)$ is the reinforcement noise.

It is trivial to show that the Bayesian update of peoples' prior $p\left(\eta \middle| \eta_G^{(i)}, \sigma_G\right)$ with noisy reinforcement signals $y^{(i)}$ yields the following posterior belief $p\left(\eta \middle| y^i, \sigma_\varepsilon, \eta_G^i, \sigma_G\right)$ about the best policy η (cf. Equation 7 in the main text):

$$p\left(\eta \middle| y^{(i)}, \sigma_\varepsilon, \eta_G^{(i)}, \sigma_G\right) = N\left(\alpha^{(i)}, \sigma^{(i)}\right) \quad : \begin{cases} \alpha^{(i)} = \sigma^{(i)}\left(\dfrac{y^{(i)}}{\sigma_\varepsilon} + \dfrac{\eta_G^{(i)}}{\sigma_G}\right) \\ \sigma^{(i)} = \left(\dfrac{1}{\sigma_\varepsilon} + \dfrac{1}{\sigma_G}\right)^{-1} \end{cases} \tag{A5}$$

where $\alpha^{(i)}$ is the agent's posterior estimate of the optimal cost-susceptibility, and $\sigma^{(i)}$ is her posterior uncertainty. By construction, the agent will arbitrate cost and benefits according to this posterior belief, i.e. $\alpha^{(i)}$ is also the agent's own cost-susceptibility.

1. The false-consensus bias

If one knew others' reinforcement signals and priors, Equation A5 could be used to form a prediction about other's cost-susceptibility. In turn, any (prior) information regarding others' reinforcement signals and priors can be used as follows.

First, let us replace $y^{(o)}$ by its definition in Equation A5:

$$\alpha^{(o)} = \sigma\left(\frac{\eta + \varepsilon^{(o)}}{\sigma_\varepsilon} + \frac{\eta_G^{(o)}}{\sigma_G}\right) \tag{A6}$$

One can derive the moments of the conditional distribution of $\alpha^{(o)}$ from priors about feedback noise $\varepsilon^{(o)} \sim N\left(0, \sigma_\varepsilon\right)$:

$$\begin{cases} E\left[\alpha^{(o)} \middle| \eta, \sigma_\varepsilon, \eta_G^{(o)}, \sigma_G\right] = \sigma\left(\dfrac{\eta}{\sigma_\varepsilon} + \dfrac{\eta_G^{(o)}}{\sigma_G}\right) \\ V\left[\alpha^{(o)} \middle| \eta, \sigma_\varepsilon, \eta_G^{(o)}, \sigma_G\right] = \dfrac{\sigma^2}{\sigma_\varepsilon} \end{cases} \tag{A7}$$

$$\Rightarrow p\left(\alpha^{(o)} \middle| \eta, \sigma_\varepsilon, \eta_G^{(o)}, \sigma_G\right) = N\left(\sigma\left(\frac{\eta}{\sigma_\varepsilon} + \frac{\eta_G^{(o)}}{\sigma_G}\right), \frac{\sigma^2}{\sigma_\varepsilon}\right)$$

One can then marginalize over others' priors $\eta_G^{(o)} \sim N\left(\Gamma_G, \Omega_G\right)$, as follows:

$$\begin{aligned} p\left(\alpha^{(o)} \middle| \eta, \sigma_\varepsilon, \Gamma_G, \sigma_G, \Omega_G\right) &= \int p\left(\alpha^{(o)} \middle| \eta, \sigma_\varepsilon, \eta_G^{(o)}, \sigma_G\right) p\left(\eta_G^{(o)} \middle| \Gamma_G, \Omega_G\right) d\eta_G^{(o)} \\ &= N\left(\sigma\left(\frac{\eta}{\sigma_\varepsilon} + \frac{\Gamma_G}{\sigma_G}\right), S\right) \end{aligned} \tag{A8}$$

where the variance $S = \sigma^2\left(\dfrac{1}{\sigma_\varepsilon} + \dfrac{\Omega_G}{\sigma_G{}^2}\right)$.

If one knew what the best policy η is, one could use Equation A8 to derive a prediction regarding the subjective preference of any individual in the population. People, however, have only uncertain (and subjective) information regarding η. Thus, marginalizing over the best policy η yields agent-dependent predictive densities:

$$\begin{aligned} p\left(\alpha^{(o)} \middle| \alpha^{(s)}, \sigma_\varepsilon, \sigma_G, \Gamma_G, \Omega_G\right) &= \int p\left(\alpha^{(o)} \middle| \eta, \sigma_\varepsilon, \Gamma_G, \sigma_G, \Omega_G\right) p\left(\eta \middle| \alpha^{(s)}, \sigma\right) d\eta \\ &\triangleq N\left(\mu_0^{(o)}, \Sigma_0^{(o)}\right) \end{aligned} \tag{A9}$$

where $\mu_0^{(o)}$ and $\Sigma_0^{(o)}$ are the first two moments of the agent's prior belief regarding any other individual's cost-susceptibility:

$$\begin{cases} \mu_0^{(o)} = \sigma\left(\dfrac{\alpha^{(s)}}{\sigma_\varepsilon} + \dfrac{\Gamma_G}{\sigma_G}\right) \\ \Sigma_0^{(o)} = \dfrac{\sigma^2}{\sigma_\varepsilon}\left(1 + \dfrac{\sigma}{\sigma_\varepsilon}\right) + \dfrac{\sigma^2}{{\sigma_G}^2}\Omega_G \end{cases} \tag{A10}$$

Now, note that Equation A5 implies $\frac{\sigma}{\sigma_\varepsilon} = \frac{\sigma_G}{\sigma_G + \sigma_\varepsilon}$ and $\frac{\sigma}{\sigma_G} = \frac{\sigma_\varepsilon}{\sigma_G + \sigma_\varepsilon}$. This can be used to express Equation A10 directly in terms of the model native parameters:

$$\begin{cases} \mu_0^{(o)} = \dfrac{\sigma_G\,\alpha^{(s)} + \sigma_\varepsilon\,\Gamma_G}{\sigma_G + \sigma_\varepsilon} \\ \Sigma_0^{(o)} = \dfrac{{\sigma_G}^2\sigma_\varepsilon}{\left(\sigma_\varepsilon + \sigma_G\right)^2}\left(1 + \dfrac{\sigma_G}{\sigma_G + \sigma_\varepsilon} + \dfrac{\sigma_\varepsilon\Omega_G}{{\sigma_G}^2}\right) \end{cases} \tag{A11}$$

This concludes the demonstration of Equation 9 in the main text.

2. The influence bias

Although at first glance counterintuitive, the influence bias is another consequence of the fact that people subjective preferences are belief about the "best" policy η. This essentially makes other's attitudes another source of information, which can be used to update one's belief.

Let us consider an agent mentalizing about an Other's attitude. After having observed t cost-benefit arbitrages, she holds the following posterior belief about the Other's cost-susceptibility (see main manuscript):

$$p\left(\alpha^{(o)} \middle| a_{\to t}, \mu_0^{(o)}, \Sigma_0^{(o)}\right) = N\left(\mu_t^{(o)}, \Sigma_t^{(o)}\right) \tag{A12}$$

This estimate can be related to the best policy η because the Other's subjective cost-susceptibility also obeys Equation A5.

First, let us consider the information one holds about η, given the other's cost-susceptibility $\alpha^{(o)}$:

$$p\left(\eta \middle| y^{(s)},\alpha^{(o)},...\right)=\frac{p\left(\alpha^{(o)}\middle|\eta,...\right)p\left(\eta\middle|y^{(s)},...\right)}{\int p\left(\alpha^{(o)}\middle|\eta,...\right)p\left(\eta\middle|y^{(s)},...\right)d\eta} \tag{A13}$$

where $p\left(\alpha^{(o)}\middle|\eta,...\right)$ simply derives from Equation A1 and $p\left(\eta\middle|y^{(s)},...\right)$ is the agent's posterior belief about η, before knowing the other's cost-susceptibility $\alpha^{(o)}$. In the following, $\alpha_1^{(s)}$ denotes the agent's cost-susceptibility before mentalizing about the other.

One can show (using simple density calculations) that, given the other's cost-susceptibility, the agent's posterior belief $p\left(\eta\middle|y^{(s)},\alpha^{(o)},...\right)=N\left(A\left(\alpha^{(o)}\right),C\right)$ about the optimal policy η is a Gaussian distribution with mean $A\left(\alpha^{(o)}\right)$ and variance C:

$$\begin{cases} A\left(\alpha^{(o)}\right)=\dfrac{C}{\sigma}\left(\dfrac{\alpha^{(o)}}{1+\dfrac{\sigma_\varepsilon\,\Omega_G}{\sigma_G{}^2}}-\dfrac{\Gamma_G}{\dfrac{1}{\sigma_\varepsilon}+\dfrac{\sigma_\varepsilon\,\Omega_G}{\sigma_G{}^2}}+\alpha_1^{(s)}\right) \\ C=\dfrac{\sigma\left(\sigma_\varepsilon\,\sigma_G^2+\sigma_\varepsilon^2\,\Omega_G\right)}{\sigma_\varepsilon\,\sigma_G^2+\sigma_\varepsilon^2\,\Omega_G+\sigma\,\sigma_G^2} \end{cases} \tag{A14}$$

Of course, Equation A14 cannot be used directly, because the other's cost-susceptibility $\alpha^{(o)}$ is not known with infinite precision. Rather, the agent's updated belief relies upon marginalizing over likely values of $\alpha^{(o)}$, as follows:

$$
\begin{aligned}
p\left(\eta\middle|a_{\to t},y^{(s)},\ldots\right) &= \int p\left(\eta\middle|a_{\to t},y^{(s)},\alpha^{(o)},\ldots\right)p\left(\alpha^{(o)}\middle|a_{\to t},y^{(s)},\ldots\right)d\alpha^{(o)} \\
&= \int p\left(\eta\middle|y^{(s)},\alpha^{(o)},\ldots\right)p\left(\alpha^{(o)}\middle|a_{\to t},\ldots\right)d\alpha^{(o)} \\
&\triangleq N\left(\alpha_2^{(s)},\sigma_2^{(s)}\right)
\end{aligned}
\tag{A15}
$$

One can show that the first two moments $\alpha_2^{(s)}$ and $\sigma_2^{(s)}$ of the updated posterior density $p\left(\eta\middle|a_{\to t},y^{(s)},\ldots\right)$ on the optimal policy η are given by:

$$
\begin{cases}
\sigma_2^{(s)} = \lambda^2\Sigma_t^{(o)} + C \\
\alpha_2^{(s)} = \alpha_1^{(s)} + \lambda\left(\mu_t^{(o)} - \mu_0^{(o)}\right)
\end{cases}
\tag{A16}
$$

where the "learning rate" λ is:

$$
\lambda = \frac{1}{\frac{\sigma_\varepsilon\Omega_G}{\sigma_G^2} + \frac{\sigma_G}{\sigma_G+\sigma_\varepsilon} + 1} \quad \Rightarrow 0 \le \lambda \le 1
\tag{A17}
$$

This completes the derivation of Equations 14-15 in the main text.

The qualitative predictions of the holistic model are described in the main text (cf. Figure 2). However, we would like to extend these analyses, and explore the predicted attitude change $\alpha_2^{(s)} - \alpha_1^{(s)}$, in response to a mismatch $\alpha^{(o)} - \alpha_1^{(s)}$ between the agent and the Other. This is depicted on Figure A1 below.

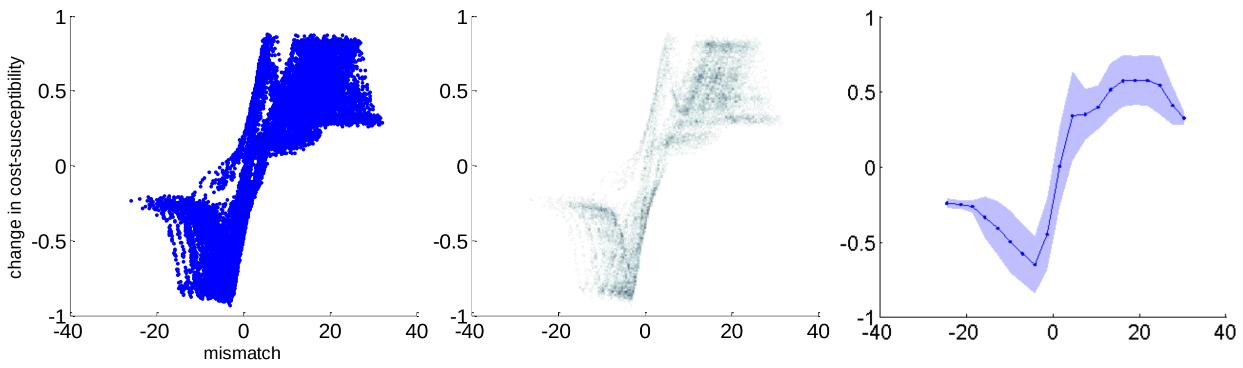

Figure A1: Qualitative predictions of the holistic model. We simulated a virtual population (endowed with arbitrary cost-susceptibilities), who learn about agents performing cost-benefit arbitrages (also endowed with arbitrary cost-susceptibilities). In all panels, the social influence bias is depicted in terms of the relationship between peoples' preference change (y-axis) and mismatch (x-axis). **Left:** each dot is one Monte-Carlo simulation (n=128×128=16,384 in total), where we sample from a broad range of cost-susceptibilities. **Middle:** same as in A, but in terms of the density of dots. **Right:** same as in A, but in terms of the mean (plain lines) and standard deviation (shaded areas) of the preference change for 20 equally-spaced bins of the mismatch.

One can see that, for intermediate mismatches, the average preference change increases monotonically with mismatch. In this intermediate regime, a unit change in mismatch $\alpha^{(o)} - \alpha_1^{(s)}$ approximately yields 10% of preference change $\alpha_2^{(s)} - \alpha_1^{(s)}$ (one order of magnitude below). However, more extreme mismatches eventually yield *decreasing* preference changes. It is as if the influence bias magnitude behaved as an inverted U-shaped function of mismatch magnitude. In other words, the holistic model predicts that when the mismatch between the agent and the Other is too high, the agent reduces his preference alignment.

III. VBA online adaptive design procedure

During both *Decision 1* and *Prediction* phases, we used an adaptive online design optimization procedure in the aim of maximizing the statistical power of post-hoc model-based data analysis. For example, the model-based analysis of trial-by-trial choice data acquired during the *Decision* phases proceeds by fitting the relevant cost-benefit arbitrage model (cf. Equations 1-4 of the main text). However, there might be an optimal manipulation of the features (e.g., reward and delay) of both choice alternatives, such that choice data yields maximal information regarding the underlying unknown parameters (i.e. the cost-susceptibility α and the behavioural temperature β). Would the cost-benefit arbitrage models be linear, the optimal sequence of choice features could be determined prior to performing the experiment. This is, however, not the case. We thus resort to an online adaptive design optimization procedure, which is tailored to the subsequent variational Bayesian data analysis (Daunizeau et al., 2014b). We summarize its rationale below.

Let u be the set of stimuli features that constitutes our design, y the data measured experimentally, and ϑ the unknown parameters of our generative model (which we denote as m). We start with the premise that the data analysis aims at deriving the posterior density $p\left(\vartheta|y,u,m\right)$ given the set of sampled measurements y. Nonlinearities in the generative model eschew exact analytical solutions to this problem, which is finessed using variational Bayesian (VB) approaches (Beal, 2003). In brief, under the Laplace approximation, VB yields a Gaussian approximate posterior $q\left(\vartheta\right)\approx p\left(\vartheta|y,u,m\right)$, whose first- and second-order moments are given by (Daunizeau et al., 2009; Friston et al., 2007):

$$q\left(\vartheta\right)=N\left(\mu,\Sigma\right):\begin{cases}\mu\approx\arg\max_{\theta} I\left(\vartheta\right)\\ \Sigma\approx-\left[\left.\dfrac{\partial^2 I}{\partial\vartheta^2}\right|_{\mu}\right]^{-1}\end{cases} \quad \text{(A17)}$$

where $I(\vartheta)=\log p(y|\vartheta,m)+\log p(\vartheta|m)$ is entirely specified by our generative model. As an example, for the *Decision* phase, the likelihood $p(y|\vartheta,m)$ is derived from inserting Equations 2-4 into Equation 1. Importantly here, diagonal elements of the posterior covariance matrix Σ measure how uncertain or vague information about the model parameters is. Note that it is an implicit function of design features ($\Sigma \equiv \Sigma(u)$), through their impact on the likelihood function. Thus, if the aim is to maximize estimation efficiency, design optimization proceeds from minimizing the trace of the expected posterior matrix w.r.t. candidate design features (cf. so-called "design A-optimality"; Myung and Pitt, 2009). At each trial t, we thus update the approximate posterior moments according to Equation A16 and chose the design features $\hat{u}_{t+1}$ such that:

$$
\begin{aligned}
\hat{u}_{t+1} &= \arg\min_{u} E\left[trace\left(\Sigma_{t+1}(u)\right)\right] \\
&\approx \arg\max_{u}\, trace\left[\left.\frac{\partial^2 I}{\partial \vartheta^2}\right|_{\mu_t}\right]^{-1}
\end{aligned}
\tag{A18}
$$

where μ_t and Σ_t are the update posterior moments. We refer the interested reader to (Daunizeau et al., 2011) for further mathematical details.

Practically speaking, the optimization in Equation A18 is performed by evaluating the A-optimality metric on a predefined 3D search grid of design features (in all conditions, one of the four features is fixed throughout the experiment; see main text). It turns out that the most informative design features are those design features that span the maximal curvature points of the sigmoid mapping of the likelihood function. This means that there are always two local design optimizers, which are likely to induce opposite behavioural tendencies. We exploit this symmetry to impose the constraint that the frequency of the Other's "impulsive" choices (low-reward/low-cost choices) is fixed to 50% during the *Prediction* phase (over the 40 trials).

IV. Supplementary results: model inversions diagnostics

1. Cost-benefit arbitrage models

Table A1 below reports the summary statistics of inversions of cost-benefit arbitrage models (given participants' behaviour in *Decision* phases 1 and 2), based upon the cost-dependent utility functions given in Equations 2-4 of the main text.

	$\hat{\alpha}$		$\hat{\beta}$		log-evidence		fit accuracy	
	Dec1	Dec2	Dec1	Dec2	Dec1	Dec2	Dec1	Dec2
Delay	1.4 (1.6)	1.2 (1.6)	.1 (.8)	.1 (.6)	-20	-18	80%	81%
Effort	-.8 (1.2)	-.7 (1.3)	.1 (1.3)	-.2 (1.1)	-17.5	-15	77%	83%
Risk	-2.5 (.9)	-2.6 (.9)	-3.0 (.8)	-3.2	-20	-18	78%	80%

Table A1. For each cost type (and each *Decision* phase), we report the group-average of estimated parameters (cost-susceptibility $\hat{\alpha}$ and behavioural temperature $\hat{\beta}$), log-evidence and balanced fit accuracy.

We also inverted a cost-benefit arbitrage model based upon a linear utility function (of the form $u_{\alpha}(R,C) = R - \exp(\alpha)C$, where *C* is the cost), which serves as a reference point for cost-benefit arbitrage models. Table A2 reports the corresponding summary statistics.

	$\hat{\alpha}$		$\hat{\beta}$		log-evidence		fit accuracy	
	Dec1	Dec2	Dec1	Dec2	Dec1	Dec2	Dec1	Dec2
Delay	-1.6 (1.2)	-1.8 (1.3)	.5 (1.2)	.3 (1.0)	-77	-73	72%	75%
Effort	.6 (1.4)	.9 (1.4)	.1 (1.4)	-.1(1.2)	-19	-17	83%	88%
Risk	-.9 (.1)	-.8(.7)	2.9 (1.3)	2.9 (1.3)	-48	-48	51%	55%

Table A2. For each cost type (and each *Decision* phase), we report the group-average of estimated parameters (cost-susceptibility $\hat{\alpha}$ and behavioural temperature $\hat{\beta}$), log-evidence and balanced fit accuracy.

One can see that for delay and risk, the linear utility model provides a less likely explanation of peoples' behaviour than the utility models given in Equation 2-4 of the main text. In fact, a group-level Bayesian model comparison largely favours the latter models against the linear utility model (exceedance probability –EP- and protected exceedance probability –PEP- larger than .999).

Although RFX-BMS still favours the nonlinear discounting model of effort (EP>.99 for both *Decision* 1 and *Decision* 2, PEP=.83 for *Decision* 1 and PEP=.95 for *Decision* 2), a fixed-effect BMS at the group-level gives inconclusive results. In addition, the fit accuracy is similarly good for both models. This suggests that, within the range of demanded efforts, the effort discounting model given in Equation 4 of the main text eventually behaves almost linearly.

Figure A2 below also reports the best and worst fit (across subjects) of utility-based arbitrage models, when inverted given participants' behaviour during *Decision* phases 1 and 2.

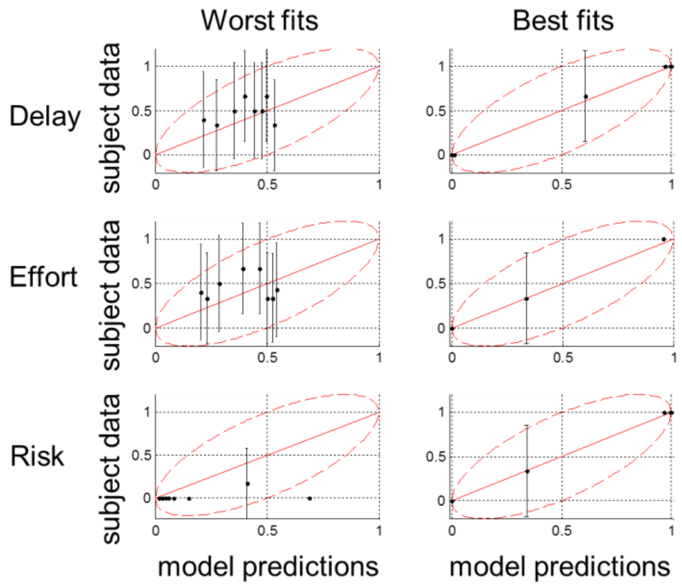

Figure A2: Worst and best fits of the cost-benefit arbitrage models for each cost type. Each graph shows the participant's observed choice data (y-axis) as a function of the model's fitted data (x-axis). Recall that the model's fitted output is the expected choice data at each trial, marginalized over model parameters. Here, the series of expected choice data was binned into eight quantiles, each of which corresponds to a subset of observed choice data with a given mean (black dots) and standard deviation (black errorbars). An ideal model fit would align along the plain red line, with errorbars matching the dashed red ellipse. **Worst fits**: Delay : fit accuracy=45%; Effort : fit accuracy=45%; Risk : fit accuracy=41%. **Best fits:** fit accuracy>99% for all cost types.

2. BPL model

The BPL was used in the main text to provide model-inspired evidence for false-consensus and influence biases. These analyses implicitly rely on the assumption that the utility functions underlying Bayesian mentalizing during the *Prediction* phase properly account for participants' intuitions about others cost-benefit arbitrages. Here, we provide evidence in favour of this assumption.

In brief, we wanted to control that (1) participants did not make predictions at random, (2) they did not use simple heuristics to derive their predictions about the Other's choice, (3) they did not always simulate their own choices during the *Prediction* phase, (4) nonlinearities in the utility functions given in

Equation 2-4 are needed to explain peoples' predictions about others. We thus performed a statistical comparison of the BPL model with the following alternative models for peoples' guesses in the *Prediction* phase:

(1) *Random model*: at each trial, the observer randomly selects his bet according to a bias δ for the low-cost option (NB: this bias can be negative).

(2) *Fictitious Play*: at each trial, the observer updates (in a Bayesian way) his estimate of the probability that the Other will choose the low-cost option (see e.g. (Devaine et al., 2014b)).

(3) *Self Preference*: at each trial, the observer's guess about the Other's choice reproduces what he would have chosen himself (this prediction can be derived from the estimated participant's cost-susceptibility during *Decision* phase 1).

(4) *Linear* BPL: at each trial, the observer the observer updates his estimate of the Other's cost-susceptibility according to Equation 7 of the main text, under a linear utility model.

When pooling evidence over cost types, RFX-BMS revealed that the nonlinear BPL was the most likely explanation to peoples' guesses during the *Prediction* phase (EP=1, PEP=1).

Figure A3 below also reports the best and worst fit (across subjects) of the nonlinear BPL model, when inverted given participants' behaviour in the *Prediction* phase.

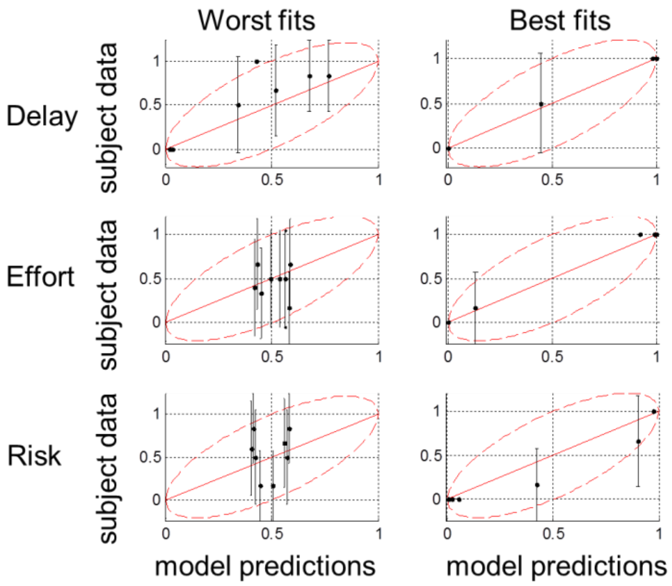

Figure A3: Worst and best fits of the BPL model for each cost type. This figure uses the same format as Figure S2. **Worst fits**: Delay : fit accuracy=65%; Effort : fit accuracy=55%; Risk : fit accuracy=55%. **Best fits:** Delay : fit accuracy>99%; Effort : fit accuracy>99%; Risk : fit accuracy=95%.

3. Bayesian model of attitude alignment

Let us first summarize the prior distributions we used to perform the Bayesian inversions of the different variants of the model. Recall that the holistic model has seven unknown parameters. The first four parameters are the sufficient statistics of peoples' priors regarding the scattering of information regarding the "best" policy, namely: σ_ε, σ_G, Ω_G and Γ_G, whose meaning is given in the main text. Here, we have re-parameterized the model, mostly in the aim of enforcing positivity constraints on variances, as follows: $\sigma_\varepsilon = \exp(\lambda_1)$, $\sigma_G = \exp(\lambda_2)\sigma_\varepsilon$, $\Omega_G = \exp(\lambda_3)\sigma_\varepsilon$ and $\Gamma_G = \lambda_4$.

The last five unknown parameters are the initial cost-susceptibility $\alpha_1^{(s)}$, the behavioural log-temperature $\beta^{(s)}$ in *Decision* phases, the log-temperature $\varphi^{(s)}$ in the *Prediction* phase as well as the sufficient statistics $\mu_0^{(o),2}$ and $\Sigma_0^{(o),2}$ describing the prior distribution over the log-temperature of the Other. Based on this model we defined 4 variants with and without false-consensus and influence biases.

The variants without influence (m_1 and m_2) simply equated peoples' cost-susceptibility in *Decision* phases 1 and 2 ($\alpha_2^{(s)} = \alpha_1^{(s)}$). The variants without false-consensus (m_1 and m_3), two parameters were added to the model: namely the prior mean $\mu_0^{(o),1}$ and variance $\Sigma_0^{(o),1}$ on the Other's cost-susceptibility $\alpha^{(o)}$ (since they are not constrained by the "holistic" model anymore).

Table A3 below summarizes the specification of the Gaussian prior distribution of these parameters, in terms of its mean and variance. Note: M_α and M_β are the sample average of peoples' cost-susceptibility and behavioural temperature (see Table A1).

Parameter	Prior Mean	Prior Variance	m_1	m_2	m_3	m_4
λ_1	0	1		X	X	X
λ_2	0	1		X	X	X
λ_3	0	1		X	X	X
λ_4	M_α	1		X	X	X
α_1^s	M_α	1	X	X	X	X
β^s	M_β	1	X	X	X	X
φ^s	-1	1	X	X	X	X
$\mu_0^{o,1}$	M_α	1	X		X	
$\mu_0^{o,2}$	M_β	1	X	X	X	X
$\Sigma_0^{o,1}$	.6	1	X		X	
$\Sigma_0^{o,2}$	.6	1	X	X	X	X

Table A3: Prior mean and variance for the parameters of the model of attitude alignment.

Note that the Bayesian model of attitude alignment yielded good fit accuracy (delay: 84%, effort: 81%, risk: 85%) in all *Decision* and *Prediction* phases. Nevertheless, for completeness, we report here its worst and best fits across subjects. These are depicted on Figure A4 below, for each cost type (same format as Figures A2 and A3).

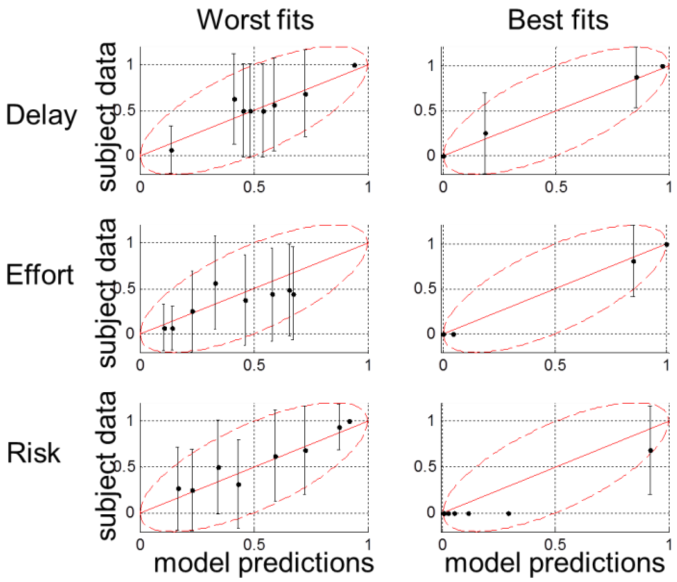

Figure A4: Worst and best fit for each cost type for the Bayesian model of attitude alignment.

References

Beal, M.J. (2003). Variational algorithms for approximate Bayesian inference /. ResearchGate.

Daunizeau, J., Friston, K.J., and Kiebel, S.J. (2009). Variational Bayesian identification and prediction of stochastic nonlinear dynamic causal models. Phys. Nonlinear Phenom. *238*, 2089–2118.

Daunizeau, J., Preuschoff, K., Friston, K., and Stephan, K. (2011). Optimizing Experimental Design for Comparing Models of Brain Function. PLOS Comput Biol *7*, e1002280.

Daunizeau, J., Adam, V., and Rigoux, L. (2014a). VBA: A Probabilistic Treatment of Nonlinear Models for Neurobiological and Behavioural Data. PLoS Comput Biol *10*, e1003441.

Daunizeau, J., Adam, V., and Rigoux, L. (2014b). VBA: A Probabilistic Treatment of Nonlinear Models for Neurobiological and Behavioural Data. PLOS Comput Biol *10*, e1003441.

Devaine, M., Hollard, G., and Daunizeau, J. (2014a). The social Bayesian brain: does mentalizing make a difference when we learn? PLoS Comput. Biol. *10*, e1003992.

Devaine, M., Hollard, G., and Daunizeau, J. (2014b). Theory of Mind: did evolution fool us? PLoS ONE *9*, e87619.

Friston, K., Mattout, J., Trujillo-Barreto, N., Ashburner, J., and Penny, W. (2007). Variational free energy and the Laplace approximation. NeuroImage *34*, 220–234.

Mathys, C., Daunizeau, J., Friston, K.J., and Stephan, K.E. (2011). A bayesian foundation for individual learning under uncertainty. Front. Hum. Neurosci. *5*, 39.

Myung, J.I., and Pitt, M.A. (2009). Optimal Experimental Design for Model Discrimination. Psychol. Rev. *116*, 499–518.
